# Modulation of α-Synuclein Fibrillation and Toxicity by 4-Phenylbutyric Acid

**DOI:** 10.1021/acschemneuro.4c00709

**Published:** 2025-02-28

**Authors:** Kristos Baffour, Neelima Koti, Tony Nyabayo, Sathvika Balerao, Carissa Sutton, David Johnson, Rishi Patel, Santimukul Santra, Tuhina Banerjee

**Affiliations:** † Department of Chemistry and Biochemistry, Missouri State University, 901 S. National Avenue, Springfield, Missouri 65897, United States of America; ‡ Molecular Graphics and Modeling Laboratory, University of Kansas, 2034 Becker Drive, Lawrence, Kansas 66018, United States of America; § Jordan Valley Innovation Center, Missouri State University, 542 N. Boonville Avenue, Springfield, Missouri 65806, United States of America

**Keywords:** α-synuclein fibrillation, amyloid protein, 4-phenylbutyric acid, molecular dynamics simulations, oligomers

## Abstract

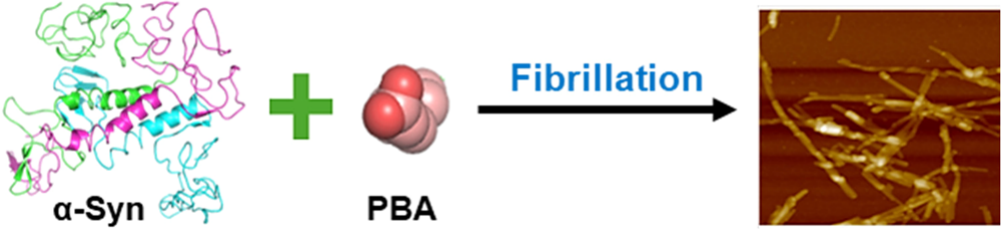

The protein misfolding and aggregation of α-synuclein (α-Syn) into neurotoxic amyloids underlies the pathogenesis of neurodegenerative diseases such as Parkinson’s disease (PD). Emerging evidence suggests that 4-phenylbutyrate (PBA) may play a role as a potential chemical chaperone for targeting α-Syn aggregation, but its molecular mechanism remains largely unknown. Using in vitro assays, we demonstrate that PBA treatment alters the pattern of α-Syn aggregation, as evidenced by reduced formation of oligomeric species and its increased susceptibility to proteolytic cleavage under the influence of PBA. Proteinase K (PK) assays, surface plasmon resonance (SPR), Nile red assays, and cytotoxicity assays indicate that PBA interacts with the extensive hydrophobic contacts of α-Syn oligomers and significantly reduces α-Syn-amyloid-induced toxicity. Furthermore, using thioflavin T-based assays, we elucidated the kinetics of PBA-mediated modulation of α-Syn aggregation, highlighting its role in accelerating the formation of α-Syn amyloid fibrils. Molecular dynamics (MD) simulations suggest PBA’s role in the destabilization of the C-terminus in α-Syn oligomers through multiple residue interactions. Collectively, our findings provide compelling evidence for the neuroprotective potential of PBA in targeting protein misfolding and aggregation in PD and suggest an avenue for disease-modifying interventions in neurodegenerative disorders.

## Introduction

Neurodegenerative diseases such as Parkinson’s disease (PD), Alzheimer’s (AD), multiple system atrophy (MSA), and prion diseases impose significant medical challenges and public health burdens on populations across the world. According to the United Nations, the incidence and prevalence of neurodegenerative diseases are expected to be 1 in 6 for the world population over 65 years by the year 2050.^1−3^ A hallmark of these diseases is the misfolding of proteins to form aggregates that causes neuronal toxicity and eventual cellular proteostatic collapse.^4−6^ There is an increased propensity for the formation of amyloid fibrils through aggregation due to the thermodynamic stability associated with extensive contacts between the chains of the protein “polymer”.^7^ Moreover, highly ordered aggregates escape the cell’s quality control systems, are difficult to degrade, and accumulate to cause cellular toxicity.^6^ Tau, αβ amyloid proteins, and synuclein proteins are some of the important intrinsically disordered proteins implicated in the disease mechanisms of neurodegenerative disorders.^8^

Alpha-synuclein protein (α-Syn), an abundant neuronal protein primarily found in the brain, has emerged as a focal point in the field of neurodegenerative research due to its central role in the pathogenesis of Parkinson’s disease (PD) and other synucleinopathies. The aggregation and misfolding of α-Syn leads to the formation of Lewy bodies and Lewy neurites which contribute to the progressive degeneration of dopaminergic neurons.^9,10^ These Lewy bodies and Lewy neurites are observed as immunopositive inclusion bodies in the brain of PD patients and manifest variations in terms of size, shape, structural characteristics, and localization.^10−14^ During the aggregation process, α-Syn generates oligomeric intermediates as it undergoes dynamic interconversion between multiple conformational states.^15,16^ As such, the formation of mature α-Syn fibrils is not a simple single-state conversion. Monomers, oligomeric intermediates, and insoluble aggregates of α-Syn are all active components of the fibrillation pathway. The mature amyloid fibrils that emerge as the terminal products of the aggregation process play a pivotal role in the prion-like dissemination of protein aggregates by recruiting native protein counterparts to adopt aberrant conformations.^17−19^ More so, the central role of α-Syn aggregation in PD is further supported by evidence that oligomeric intermediates of α-Syn are the most potent neurotoxic species in the fibrillation pathway and cause cell death in PD.^17,20^ However, the transient and heterogeneous nature of α-Syn oligomers makes it difficult to study the intricate relationship between their structure and toxicity. It is crucial to study α-Syn-related pathology to halt the progression of PD.^17,21−23^

The precise triggers for α-Syn misfolding are not fully understood, but various factors, including genetic mutations, post-translational modifications, oxidative stress, and interactions with other cellular components, have been implicated in promoting this process.^24−26^ The idea that “pathological” or aberrant aggregation originates from a crucial partially folded intermediate precursor has been reported in the literature.^27,28^ Large nonpolar patches (contiguous hydrophobic side chains) on the surface of α-Syn intermediates may cause hydrophobic contacts between molecules, which in turn could cause intermolecular interactions and aggregation. The process begins with the nucleation phase, where a small population of α-Syn molecules undergoes conformational changes and forms initial aggregates or seeds.^29−31^ These seeds can serve as templates for further aggregation and formation of oligomers and polymorphic fibrils. It has been reported that α-Syn variants with a high propensity to form oligomers in rat brains had greater neuronal toxicity and increased cell mortality than the variants that primarily formed amyloid fibrils.^32,33^

In recent years, chemical chaperones have gained increasing attention for their potential to mitigate protein misfolding, aggregation, and subsequent cytotoxicity, all without being compromised. By interaction with misfolded or partially folded protein intermediates, chemical chaperones can facilitate proper protein folding and inhibit the formation of toxic protein aggregates. In addition to this, it has been reported that chemical chaperones tend to activate cellular stress response pathways, such as the unfolded protein response (UPR) and the heat shock response (HSR), which are crucial for coping with protein misfolding and maintaining cell viability under stressful conditions.^34,35^ For instance, curcumin and NAC peptides are known to inhibit the formation of α-Syn aggregates in vitro and attenuate the toxicity of α-Syn aggregates in cells.^36−38^ However, the therapeutic potential of these drugs is challenged by issues such as poor bioavailability, peripheral toxicity due to off-target effects, interference with other protein aggregates, and the need for higher concentrations to achieve relevant toxicity during preclinical trials.^38−40^

An FDA-approved drug, 4-phenylbutyric acid (PBA), is a short-chain fatty acid that can be taken orally and used as a chemical chaperone to treat a variety of illnesses. These ailments include cystic fibrosis, homozygous β-thalassemia, spinal muscular atrophy, and illnesses related to urea metabolism.^41^ Furthermore, PBA has been shown to display neuroprotective characteristics in experiments conducted in cell cultures that are potentially mediated by suppressing histone deacetylases. In PD, the oral administration of PBA to the mouse model indicates that PBA is not neurotoxic to human α-Syn immunopositive neurons. Moreover, PBA was found to attenuate the loss of dopaminergic neurons in the substantia nigra, resulting in the improvement of motor function in the murine models. Yet the exact mechanism of PBA interaction with α-Syn during fibrillogenesis remains to be fully characterized. Understanding the mechanisms by which this molecule interacts with α-Syn and the impact on its structural and functional properties is crucial for restoring protein function and preventing pathological protein aggregation. To understand how PBA modulates the fibrillation phenomena, we assessed the binding interactions, structural shifts in the protein, and morphology of the resulting fibrils using a combination of biophysical techniques and microscopic analysis. Further, we have attempted to explore the molecular interactions between PBA and α-Syn oligomers using MD simulations.

## Results and Discussion

### Kinetics of Fibril Formation Using the Thioflavin T (ThT) Kinetic Assay

**Figure 1:**
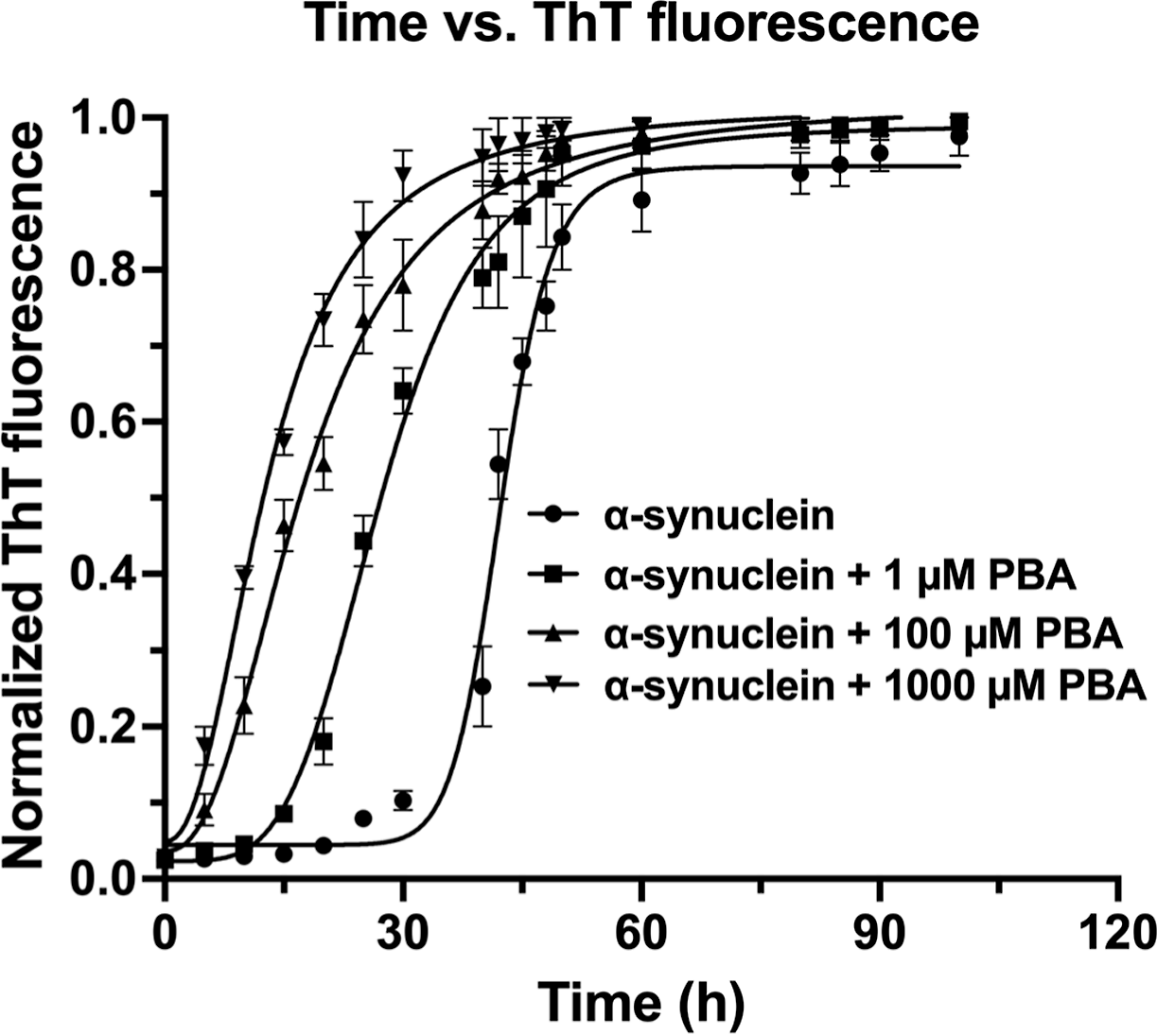
Aggregation kinetics profile of α-synuclein in the absence (control: α-synuclein) and presence of 1 μM, 100 μM, and 1000 μM PBA concentrations, showing the sigmoidal curve of aggregation indicative of nucleation-dependent polymerization. The graph shows means ± SD (*n* = 3 replicates).

In our initial studies, the self-assembly behavior of α-Syn in the absence and presence of PBA was determined using thioflavin T (ThT) binding assays. ThT is a fluorescent probe known to bind alongside the side chain grooves of amyloidogenic β-rich strands perpendicular to the fibril axis. Upon interaction with amyloid aggregates, ThT shows a characteristic red shift of its emission spectrum including an enhanced fluorescence at ∼482 nm when excited. This establishes ThT as a preferential and reliable marker for the detection of amyloid formation. At 0 h incubation, ThT fluorescence data indicates that there is minimal ThT binding for all samples including α-Syn without PBA (control) and α-Syn with PBA (Figure 1). The kinetics of α-Syn fibrillation proceeded through a nucleation-dependent step, and hence, the fluorescence data was fitted to a sigmoidal plot. We determined the reaction rate constant, half-time (*t*_1/2_)—the time at which ThT fluorescence reaches 50% of the maximum amplitude, and the lag time (*t*_lag_) at which ThT fluorescence reaches 5% of the maximum amplitude (Table S1). The introduction of 1 μM, 100 μM, and 1000 μM PBA resulted in a significant decrease in both lag times and half-life values. The altered kinetics suggests that even a low concentration of PBA has a modulating effect on α-Syn fibrillation and can possibly influence nucleation or elongation phases. The increase in PBA concentration correlates with a more pronounced decrease in the lag phase, which corresponds to a faster progression to the saturation step and maturation of oligomers. These findings suggest a concentration-dependent effect with higher PBA concentration modulating the fibrillation via a shorter lag phase and increased kinetic rate. The decrease in the lag time implies a quicker onset of fibrillation. The decrease in half-life and increase in kinetic rate values indicate that a short duration of time is required for α-Syn maturation under the influence of PBA.

Knowing that the PBA alters the kinetics of α-Syn fibrillation through a reduced lag phase, we desired to understand the effect of PBA on the seeding capacity of α-Syn fibrils. Seeding experiments were performed in the presence of (i) α-Syn fibrils (control) and (ii) α-Syn fibrils formed in the presence of PBA (5% seeds). In the absence of PBA, α-Syn fibrillation demonstrated a sigmoidal pattern with a significantly decreased lag phase. However, seeds formed in the presence of PBA exhibited a more pronounced effect on the fibrillation kinetics of α-Syn compared to the effect of α-Syn seeds without PBA. α-Syn seeds formed in PBA modulated α-Syn fibrillation kinetics through a concentration-dependent effect, with the seeds formed in the presence of 1000 μM PBA significantly accelerating aggregation kinetics (Figure S1).

### Biophysical Characterization of α-Syn Fibrils Using Congo Red and Nile Red Assays

**Figure 2:**
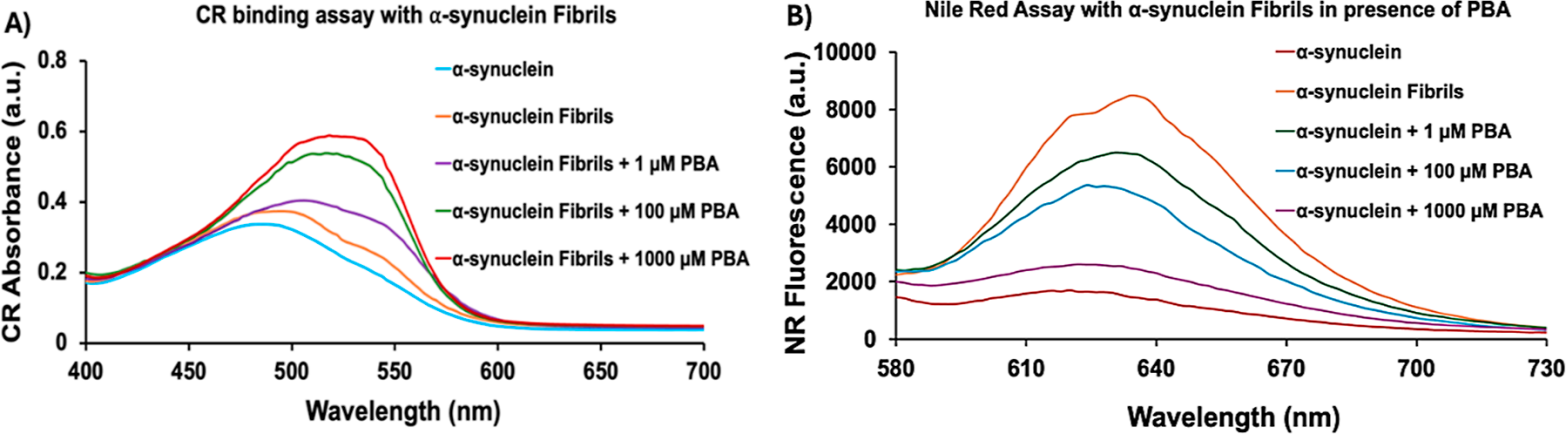
(A) Congo red absorption and (B) Nile red fluorescence in the absence and presence of 1 μM, 100 μM, and 1000 μM PBA concentrations.

With the evidence that PBA induced an early maturation of α-Syn, Congo red (CR) assays were performed to further characterize the fibrillar α-Syn aggregates in the absence and presence of PBA. CR is a distinctive marker for the presence of amyloid fibrils and exhibits a shift in absorbance from 490 to 540 nm. The CR signal of monomeric α-Syn displays an absorbance maximum at ∼488 nm at a lower intensity indicative of the absence of amyloidogenic species (Figure 2A). After the formation of α-Syn fibrils, the spectrum shows a slight increase in both CR absorption maximum and wavelength, suggesting an interaction between CR molecules and β-rich sheet α-Syn fibrillar aggregates. The CR absorption of α-Syn in the presence of PBA demonstrated a concentration-dependent profile with the highest intensity and red shift observed in 1000 μM PBA. The broader peaks seem to suggest the presence of CR-bound fibrils and possibly unbound CR molecules in the samples. Regardless, the data presented here confirm the increase in the propensity to form β-rich sheet α-Syn amyloids induced by increasing concentrations of PBA.

To assess the exposure of the hydrophobic surface to α-Syn fibrils formed in the presence and absence of PBA, we conducted Nile red (NR) fluorescence analysis. Previous studies have demonstrated that NR fluorescence increases severalfold when it binds to the exposed hydrophobic surfaces of protein aggregates. In comparison to monomeric α-Syn, an increase in NR fluorescence intensity was observed upon incubation with α-Syn fibrils (Figure 2B). The increased fluorescence indicates the interaction of NR with the exposed hydrophobic regions on α-Syn fibrils. α-Syn fibrils formed in the presence of PBA exhibited reduced NR fluorescence. Consequently, the decline in the fluorescence intensity of α-Syn fibrillar aggregates in the presence of increasing concentrations of PBA indicates a diminished level of exposed hydrophobic surfaces. This illustrates PBA’s ability to bind to the hydrophobic regions of α-Syn fibrils, reducing their accessibility to NR which subsequently decreases NR fluorescence intensity. Among the different concentrations of PBA, 1000 μM exhibited the greatest reduction in NR fluorescence intensity, with an intensity significantly lower than that of α-Syn fibrils.

### Conformational Changes in α-Syn in the Presence of PBA Using CD Spectroscopy

**Figure 3:**
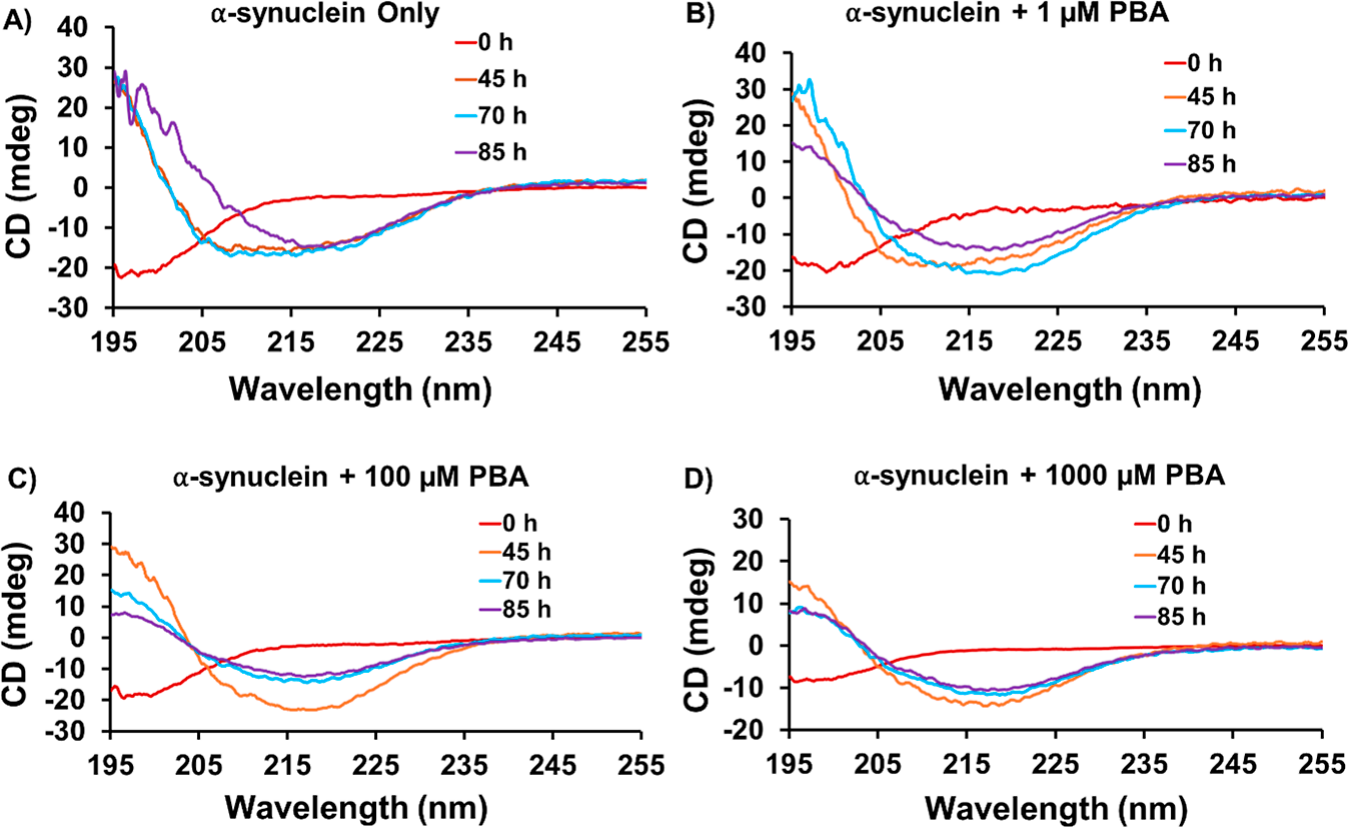
Far UV-CD spectra of α-Syn at different time intervals and concentrations of PBA: (A) α-Syn only, (B) α-Syn +1 μM, (C) 100 μM, and (D) 1000 μM PBA.

To further investigate the conformational properties of α-Syn in the presence of PBA, circular dichroism (CD) experiments were performed at different time points. In the control experiment, the secondary structure of α-Syn was monitored at four different time points: 0, 45, 70, and 85 h (Figure 3A). Consistent with the literature, secondary structural transitions show that α-Syn at 85 h assumes the β-rich sheet structure after passing through an intermediate conformation different from the characteristic random coil conformation observed at the start of the experiment. Different concentrations of PBA (1 μM, 100 μM, and 1000 μM) were incubated with the α-Syn monomer under fibrillation conditions. At 0 h, the CD data for three concentrations show an unstructured conformation which corresponds to a random coil structure (Figure 3B–D). At the concentration of 1 μM PBA, there are only modest changes to the CD with a slight broadening of the peaks corresponding to α-helical conformation when compared to the α-helical signal observed in the control at the time point of 45 h. However, for the same concentration of PBA, we observe a transition from an α-helical structure to a β-rich sheet structure in contrast to the prior α helical structure in the control at the time point of 70 h (Figure 3A,B). At 100 μM, the three time-point 45, 70, and 85 h samples are observed to have a negative minimum (∼218 nm), consistent with the β-rich sheet structure (Figure 3C). As the concentration of PBA increases, a noticeable acceleration is observed in the α-helical to β-sheet transition in contrast to that of the control (Figure 3B–D). In both Figure 3C,D, the α-helical structure was not observed, and a direct transition from random coil to β-sheet conformation during fibrillation was evident. Figure S9 shows an overlay of the Far-UV CD spectra of α-Syn at 85 h. These experimental findings were also supported by the analysis of the secondary structural content using BeStSel software as summarized in Table S3.^42^ In addition, SDS-PAGE experiments of aggregated α-Syn samples at 85 h in the absence and presence of 1000 μM PBA following ultracentrifugation (Figure S10) indicated the presence of oligomeric intermediates with various sizes.

### Characterization of α-Syn-PBA Interactions Using Surface Plasmon Resonance (SPR)

**Figure 4:**
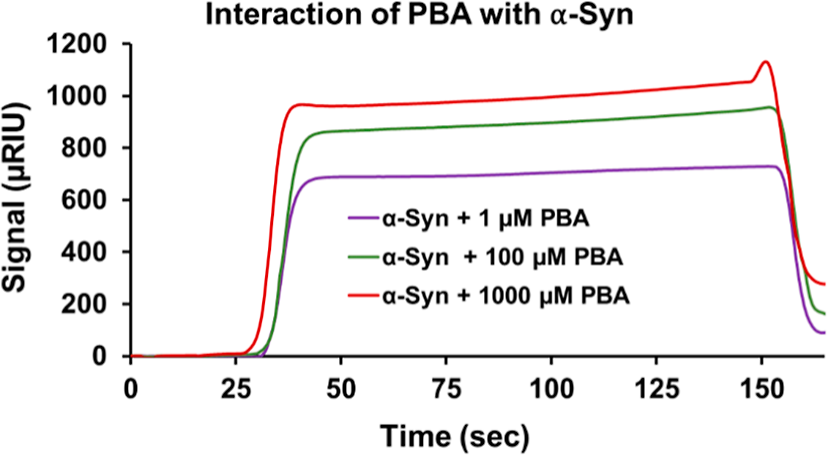
SPR sensorgram illustrates the binding interaction of PBA with α-Syn.

To obtain mechanistic insights on how PBA interacts with the α-Syn monomer, SPR experiments were performed. The α-Syn monomer was immobilized on the CM5 sensor chip, and different concentrations of PBA were injected. As PBA molecules interact with the immobilized monomer on the chip surface, changes in the refractive index at the surface occur, leading to changes in the SPR signal. The SPR response curve of α-Syn with 1 μM of PBA showed the lowest response, indicating a weaker binding interaction with the monomer (Figure 4). The highest response and strong binding interaction was recorded for the α-Syn monomer with 1000 μM PBA. Ultimately, the results indicated a concentration-dependent binding interaction between the α-Syn monomer and PBA. Subsequently, our SPR results affirm a direct binding relationship between PBA and α-Syn and rule out significant solvent interactions that could mediate α-Syn behavior in the presence of PBA.

### Assessment of Stability of α-Syn Fibrils Using Proteinase K Digestion Assays

**Figure 5:**
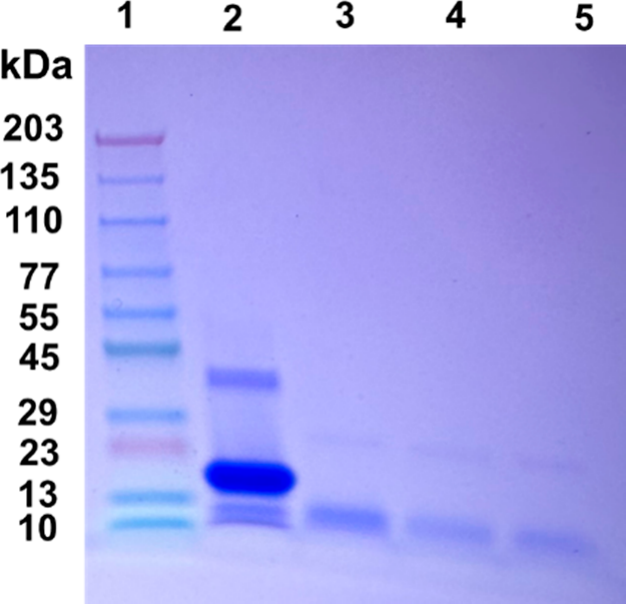
SDS PAGE analysis of the proteinase K digestion assay of α-Syn fibrils in the presence and absence of PBA. (1) Ladder, (2) α-Syn fibrils with proteinase K, (3) α-Syn fibrils in the presence of 1 μM PBA and proteinase K, (4) α-Syn fibrils in the presence of 100 μM PBA and proteinase K, and (5) α-Syn fibrils with 1000 μM PBA and proteinase K.

To examine the stability of the fibrils generated in the absence and presence of PBA, PK digestion assays were conducted. SDS PAGE analysis of the digestion reveals amyloidogenic components of α-Syn present in the sample with and without PBA. The digestion of fully formed α-Syn fibrils with PK shows multiple bands that correspond to a trimeric α-Syn species (∼46 kDa), a dimeric intermediate (∼27 kDa), and fragments of α-Syn (∼7 kDa) as shown in Figure 5, Column 2. On the contrary, PK digestion of α-Syn fibrils in the presence of known PBA concentrations of 1 μM, 100 μM, and 1000 μM shows a digestion pattern that lacks both the trimeric and the dimeric intermediate bands (Figure 5, columns 3–5). The lack of these two bands suggests that α-Syn oligomers are more prone to PK cleavage in the presence of PBA. This observation is further supported by PBA interaction with the hydrophobic core of α-Syn as observed in the NR studies. The noticeable dimeric and trimeric bands in the PK assay of α-Syn fibrils (without PBA) denote a proteolytic-resistant hydrophobic core, which is cleaved only after PBA interaction with the fibrils (Figure 5, column 2). It has been shown in previous studies that PK-resistant fragments of α-Syn fibrils are consistent with sequences from the NAC (central hydrophobic region) portion of α-Syn. The extent of proteinase K digestion was similar in the α-Syn samples incubated with different PBA concentrations.

### Morphological Analysis of α-Syn in the Presence of PBA

**Figure 6:**
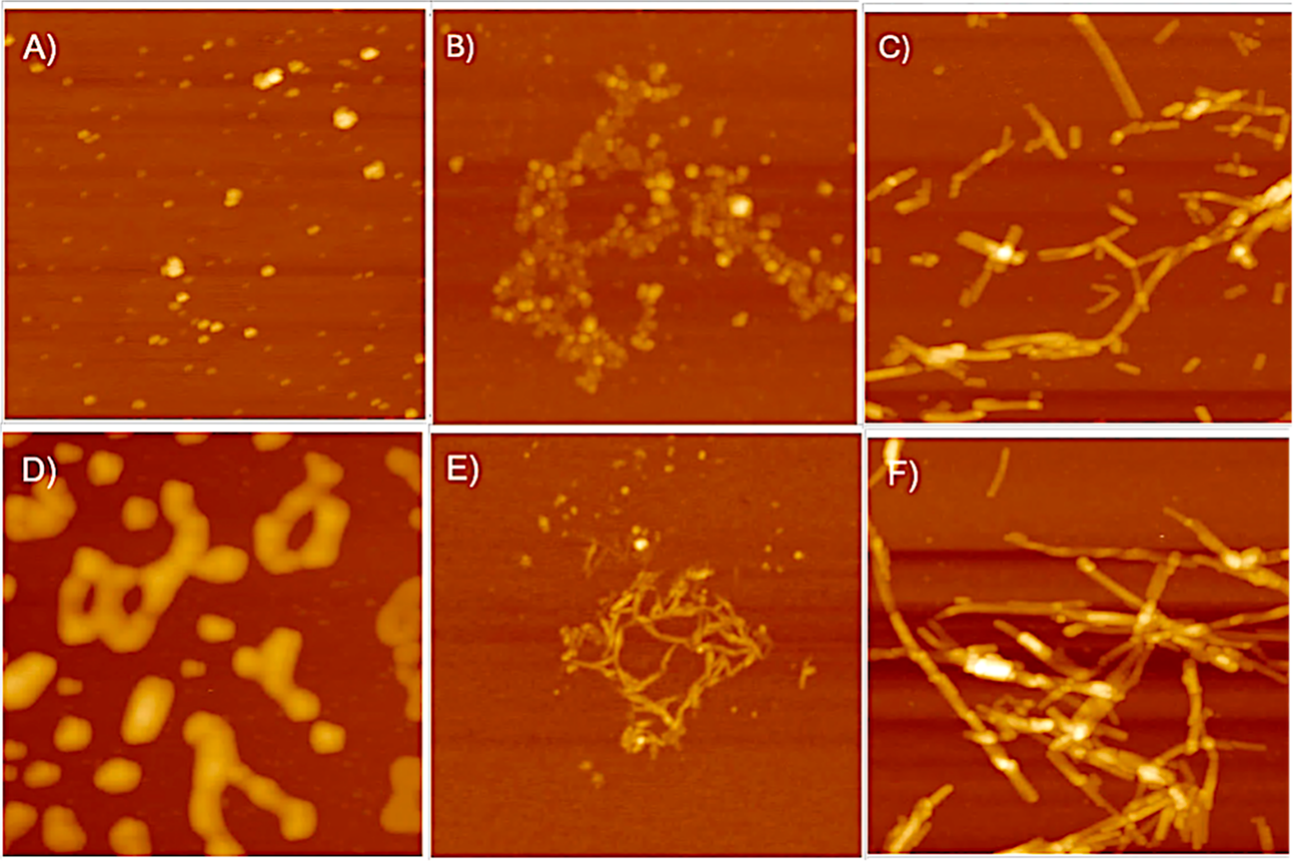
Representative AFM images showing the morphology of α-Syn fibrils at different time intervals at the scale of 1 μm. (A) α-Syn at 12 h, (B) α-Syn at 45 h, (C) α-Syn at 85 h, (D) α-Syn + 1000 μM PBA at 12 h, (E) α-Syn + 1000 μM PBA at 45 h, and (F) α-Syn + 1000 μM PBA at 85 h.

To understand the morphological changes PBA has on α-Syn fibrillar aggregates, atomic force microscopy (AFM) was used to obtain visual images of aggregates formed at the end of fibrillation. A PBA concentration of 1000 μM was chosen for this experiment as it produced the most noticeable effect and yielded a sharp transition in the secondary structure as compared to the control. AFM images of α-Syn aggregates in the absence of PBA, at a time point of 12 h, showed low-order globular aggregates of diameter ∼0.4–1.2 nm. Isolated samples studied in SDS PAGE reveal a molecular weight of dimeric and trimeric forms of α-Syn and thus may possibly correspond to α-Syn oligomeric intermediates. In contrast, when α-Syn was incubated with 1000 μM PBA, the aggregates were rather larger and elongated globular entities at a 12 h time point (Figure 6A,D). When similar experimental conditions are performed, however, at a time point of 45 h, AFM images reveal that varied species are formed during the fibrillation. At 45 h and in the absence of 1000 μM PBA, α-Syn aggregates observed are more clustered, chainlike, and spheroidal (Figure 6B). In the presence of 1000 μM PBA, the morphology changes from a nonfibrillar, clustered, chainlike spheroidal form to elongated filamentous aggregates (Figure 6E). At time point 85 h, fibrillar forms of α-Syn, mostly of height ∼2.3–5.1 nm, are observed in the absence of PBA (Figure 6C). In the presence of PBA, the fibrils are observed to be more elongated with a notable increase in the degree of clustering and length of fibrils, with a height of ∼9.1–10.4 nm (Figure 6F). The observed low-order globular α-Syn and chainlike, clustered, spheroidal α-Syn may correspond to oligomers with the short fibrillar forms, agreeing with protofibril morphology, while the longer filamentous aggregates due to increased linking among those aggregates would correspond to α-Syn fibrils. Noteworthily, the results indicate that PBA interaction with α-Syn altered their morphology from low-order species to high-order aggregates. We also observe a significant increase in the propensity to form longer fibrillar structures.

**Figure 7:**
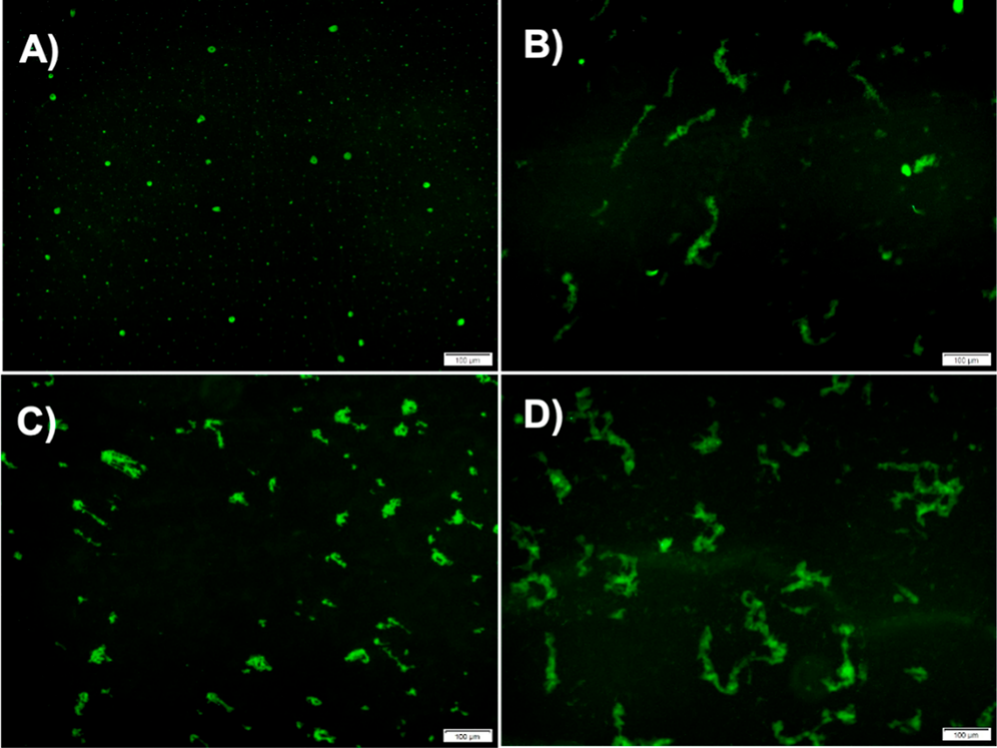
Characterization of α-Syn fibrils using fluorescence microscopy at different time points at a scale of 100 μm: representative fluorescence images (A) in the absence of PBA at 45 h, (B) in the presence of 1000 μM PBA at 45 h, (C) in the absence of PBA at 85 h, and (D) in the presence of 1000 μM PBA at 85 h.

To further confirm the morphological changes PBA induces during α-Syn fibrillation, we obtained fluorescence images of the α-Syn aggregates under specific conditions. In the absence of PBA, α-Syn aggregates at 45 h are observed as tiny green circular fluorescence spots (Figure 7A). However, under the conditions of 1000 μM PBA, α-Syn aggregates at 45 h are observed to form elongated fibrillar structures (Figure 7B). This transition in morphology is consistent with the observation of low-order globular α-Syn (in the absence of PBA) and larger and elongated globular entities (in the presence of PBA) using AFM (Figure 6A,D). α-Syn aggregates at 85 h in the absence of PBA are observed as populated with elongated structures (Figure 7C). In contrast, under the influence of 1000 μM PBA, the structures observed are more broad, elongated, and filamentous with an increase in length (Figure 7D). Like our observations from the AFM images, we observe short fibrillar forms of α-Syn transition into longer fibrils with an increase in the degree of clustering. Such observations further augment our understanding of PBA as a small molecule that makes α-Syn aggregates susceptible to the formation of longer fibrillar entities in the fibrillation pathway. More so, a comparative analysis of these fluorescence images supports our initial observation that PBA induces and accelerates the formation of filamentous aggregates in α-Syn.

### Evaluation of Polydispersity and Size Distribution of α-Syn

**Figure 8:**
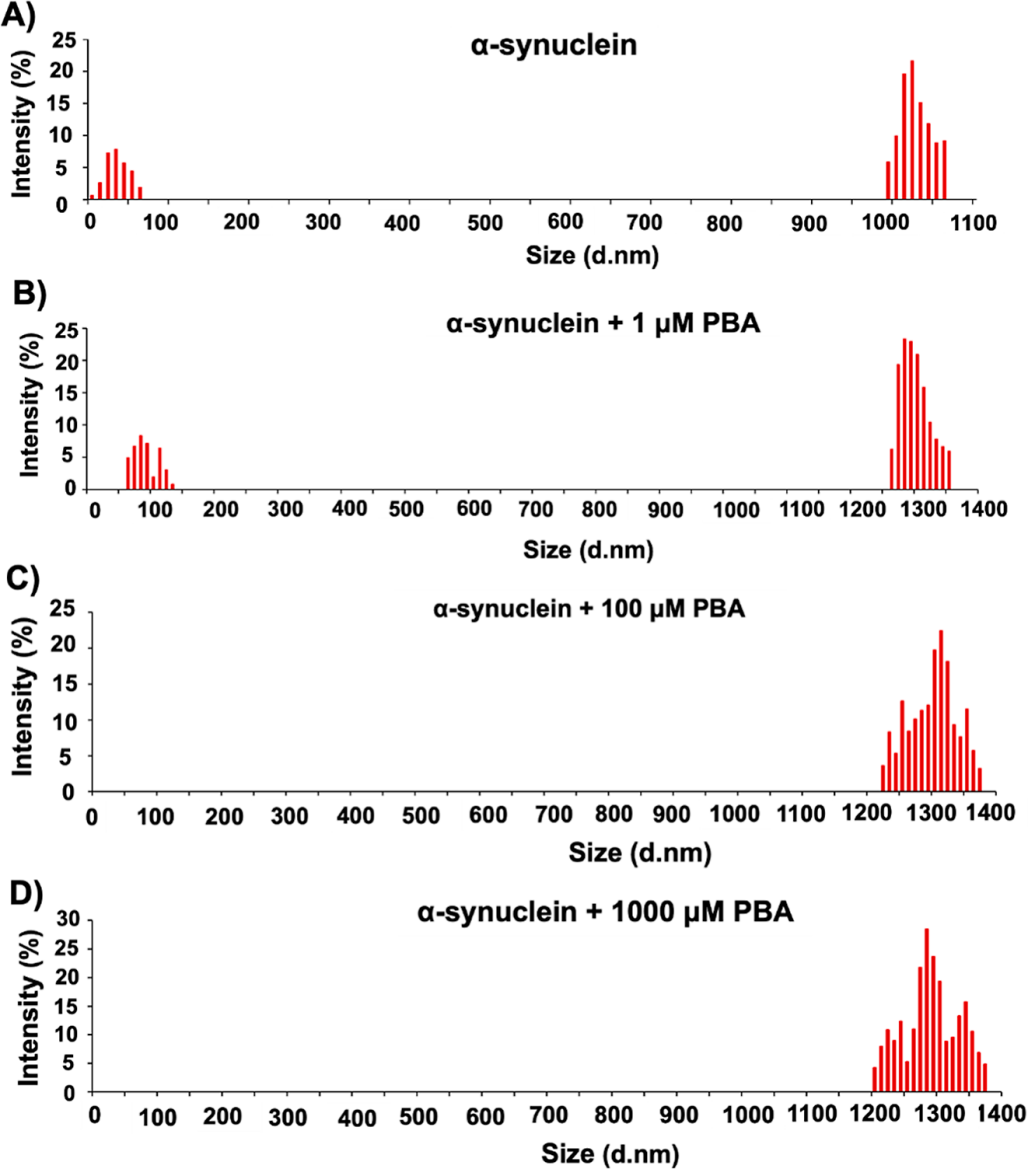
Representative DLS profiles of (A) α-Syn in the absence of PBA and in the presence of PBA at (B) 1 μM, (C) 100 μM, and (D) 1000 μM concentrations at 85 h.

Given that PBA altered the morphology of α-Syn in a manner that produced aggregates of different sizes, DLS was used to assess the size and polydispersity of α-Syn aggregates formed in the presence of PBA at a time point of 85 h. The size of the aggregates and the degree of polydispersity can be quantified by analyzing the intensity and breadth of the intensity autocorrelation function. Changes in polydispersity may indicate alterations in the uniformity or heterogeneity of the aggregates. Figure 8 reports the histograms of intensity for α-Syn alone (Figure 8A) and the samples incubated with different concentrations of PBA (Figure 8B–D). α-Syn without PBA shows two regions of different intensity, indicating that the α-Syn fibrils may contain different-sized aggregates of the protein (Table S2). The different sizes of the aggregates predicted support our observation from AFM given the varied population of aggregates observed. Under the condition of α-Syn incubated with PBA, the lack of lower particle size and consistently large particle size at even lower concentrations of PBA indicate the formation of larger aggregates. This observation supports the formation of longer and larger α-Syn fibrils as evident in the increased extent of linking between aggregates, as observed in the AFM images (Figure 6E,F). In addition, we observe a relatively broad distribution band in the higher-order fibrils present in samples incubated with PBA. This broad distribution is a measure of polydispersity of α-Syn, indicating a varied fibril length among aggregates incubated with PBA.

### Determination of α-Syn Cytotoxicity in the Presence of PBA

**Figure 9:**
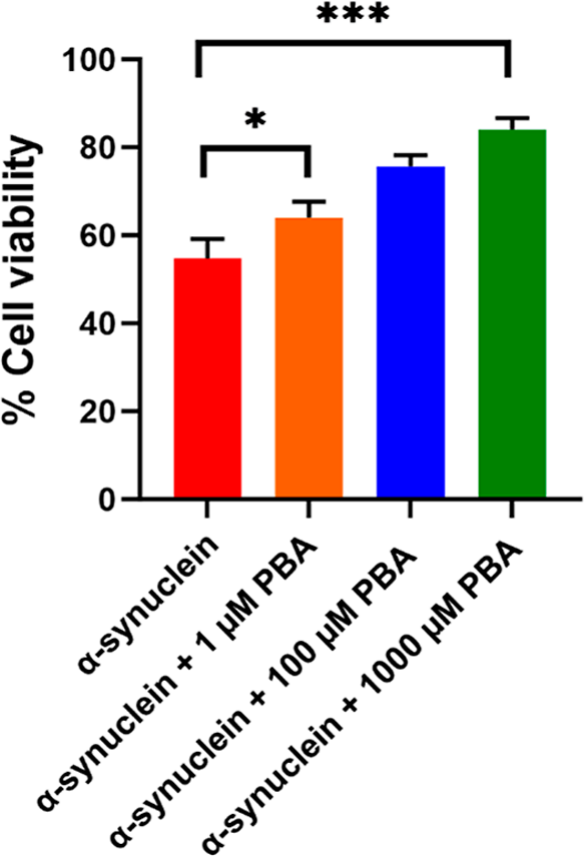
MTT assay of α-Syn in the absence (control) and presence of 1 μM, 100 μM, and 1000 μM PBA concentrations. For the MTT assay, ± SD was calculated from the average of 3 wells. Statistical data analysis was performed using one-way ANOVA, and Dunnett’s post-hoc test was employed for multiple comparison; *P < 0.05 and ***P < 0.001 indicate statistically significant differences.

To explore the influence of PBA on α-Syn amyloid-induced cytotoxicity, cell viability experiments utilizing MTT assays were conducted with SH-SY5Y neuroblastoma cells. The principle behind the assay involves the reduction of MTT to formazan crystals by mitochondrial dehydrogenases present in metabolically active cells. These formazan crystals can be solubilized and quantified spectrophotometrically with the intensity of the color directly proportional to the number of viable cells. In this study, three concentrations of PBA (1 μM, 100 μM, and 1000 μM) and the control were assessed for α-Syn-fibril-mediated cytotoxicity. As a control, cell viability studies were performed using PBA only which showed minimal toxicity. Co-incubation of α-Syn with increasing concentration of PBA in SH-SY5Y cells demonstrated decreased toxicity as depicted in Figure 9. Notably, significant cell viability was observed in the presence of 1000 μM PBA in comparison to α-Syn fibrils alone. The increase in cell viability observed in the presence of PBA is indicative of a significant reduction of the effect of α-Syn oligomers, which have been posited to be the most neurotoxic agents in the fibrillation pathway. The MTT results further augment the ThT findings of the PBA-accelerated elongation step for the maturation of α-Syn fibrils.

### Modeling of the Interaction of PBA with Helical Oligomers

**Figure 10:**
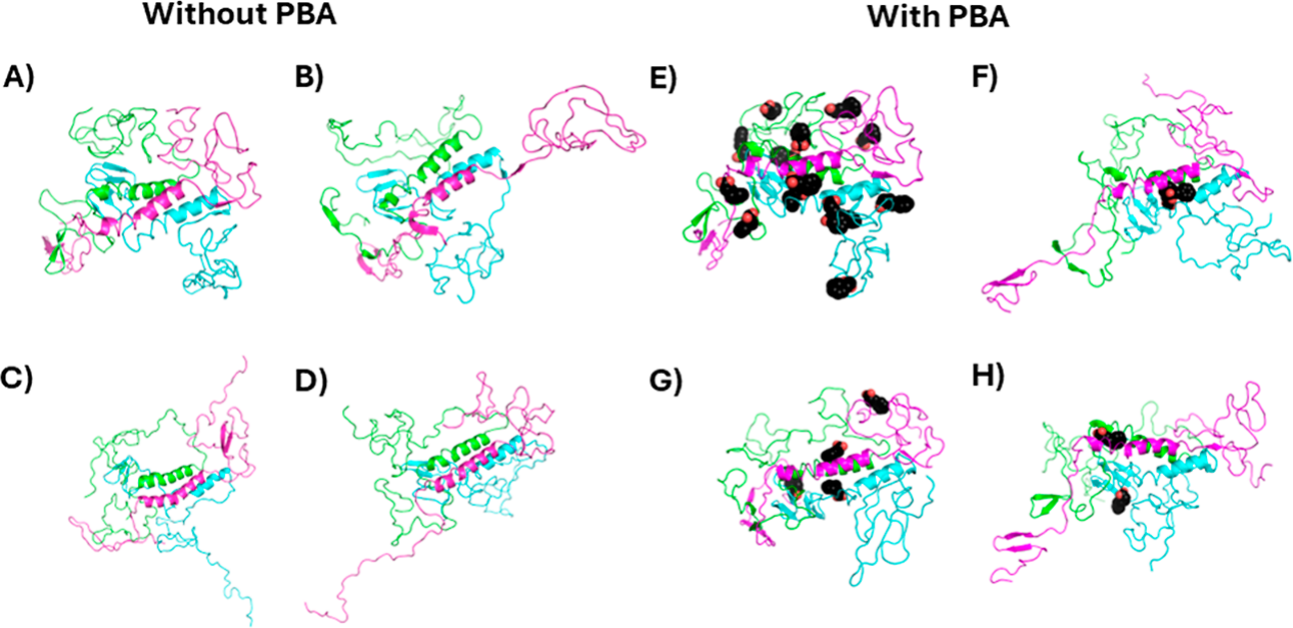
Initial and final conformations of the 25 ns molecular dynamics (MD) simulations. Initial (A) and final (B–D) conformations of unbound trimeric α-Syn and initial (E) and final (F–H) conformations of α-Syn with PBA are shown. Each α-Syn chain is represented as a different-colored cartoon, while PBA molecules are shown as black spheres.

To formulate a more detailed hypothesis of the effect of PBA on α-Syn fibrillation in the presence of PBA, molecular dynamics simulations were performed on α-Syn helical oligomers in the presence of PBA. Gurry et al. had previously generated a set of trimeric and tetrameric α-Syn oligomers.^43^ We selected a helical-rich trimer and tetramer structure and performed three simulations of a 25 ns simulation in the presence and absence of PBA molecules. The selected trimer structure featured an offset bundle of helices composed of residues 52–64, an N-terminus with two small beta hairpins and one small β sheet (bottom left corner), and disordered NAC and C-termini domains (top and right sides), as shown in Figure 10A. Global docking using Autodock Vina identified 14 sites where docked PBA molecules clustered (Figure 10E). Twenty-five ns were performed to assess the short-term effects of PBA, and the system showed stability with pressure during the *NVT* equilibration phase (Figure S2) and with density during the *NPT* production phase (Figure S3). Following the 25 ns MD simulation, the unbound trimer had some changes in the C-termini, including a crankshaft motion or partial unfolding in the magenta chain and stretching of the green and cyan chains (Figure 10F). After 25 ns in the presence of PBA molecules, all but 2–4 disassociated from the protein (Figure 10B–D). Two of the three replicants had a shared binding site on the helical bundle (Figure 10B,C) and the N-terminal β sheet (Figure 10C,D). In terms of the overall structure of the trimer in the presence of PBA, once, one of the N-termini extended outward, while the C-termini were perhaps stretched out a little but generally remained in a molten globule.

**Figure 11:**
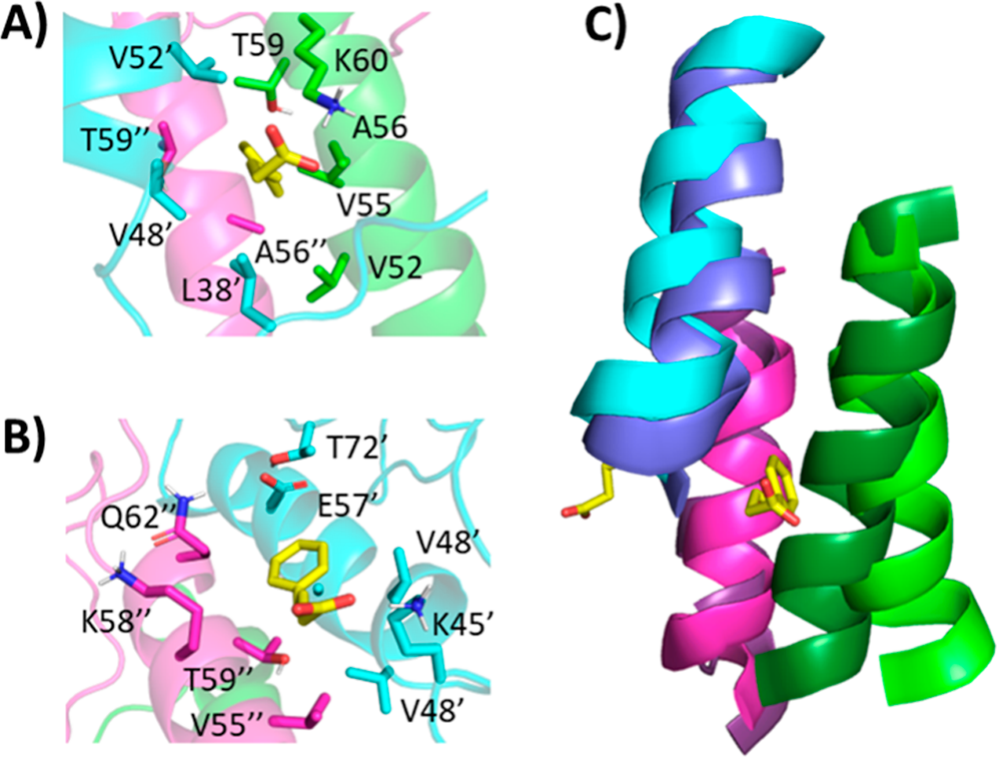
Binding of two PBA molecules (A,B) to trimeric α-Syn following 25 ns MD simulation replicant 1. Each α-Syn chain is represented as a differently colored cartoon, while PBA molecules are shown as salmon spheres. (C) The orientation between the trihelical bundles following 25 ns for unbound (darker shades) and PBA-bound (lighter shades) systems.

Further investigation of the interaction of the two PBA molecules with the first replicant yields some interesting observations. In both cases, the aromatic ring finds a hydrophobic pocket, while the carboxylate interacts with a nearby lysine. Strikingly, one PBA molecule inserted its aromatic ring into a hydrophobic cleft between the three helices, with the carboxylate interacting with Lys60 (Figure 11A). The other PBA molecule was located with the aromatic ring in a hydrophobic cleft between two helices and the carboxylate interacting with Lys45 (Figure 11B). The difference between the trihelical bundle following 25 ns in the presence and absence of PBA is highlighted in Figure 11C. The insertion of the aromatic ring between the helices causes them to angle apart relative to their conformation without PBA. However, the 25 ns time scale was not enough to assess whether PBA destabilizes the trimers, promoting fibrillation.

**Figure 12:**
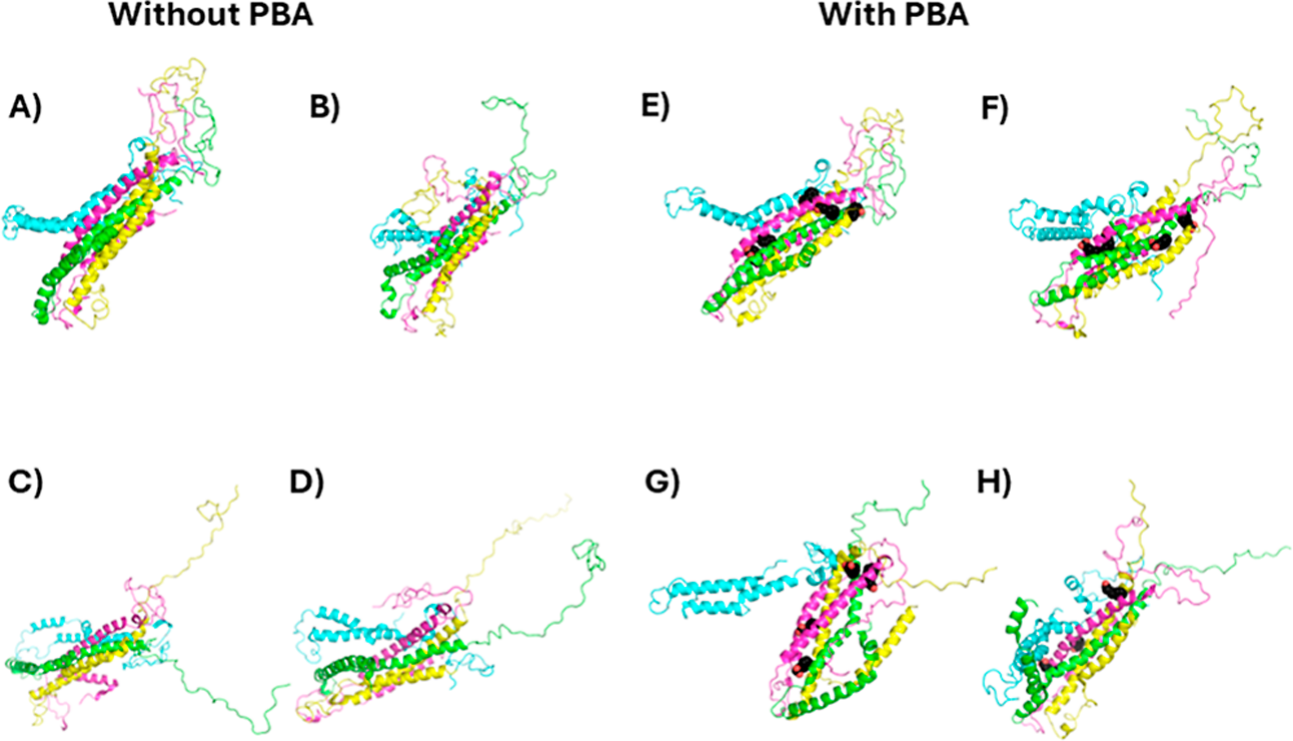
Initial and final conformations of the 25 ns molecular dynamics (MD) simulations. Initial (A) and final (B–D) conformations of tetramer α-Syn unbound and initial (E) and final (F–H) conformations of α-Syn with PBA are shown. Each α-Syn chain is represented as a different-colored cartoon, while PBA molecules are shown as black spheres.

With the tetrameric structure, however, PBA induced a striking change that could result in the eventual disassociation of the tetramer and promote fibrillation. The selected tetramer structure featured four central sets of helices from residues 56–99, surrounded by N-terminal helices from residues 5–32 (Figure 12A). Such a structure has the entire NAC domain in the helical bundle, which, logically, prevents fibrillation. The disordered C-termini forms a well-packed globular structure (top right) which could help hold the helical NAC domain bundle together on its C-terminal end. Global docking only identified 5 sites where docked PBA molecules clustered, 3 of which were located at the C-terminal half of the NAC domain and the other 2 on the N-terminal half of the NAC domain (Figure 12E). Following the 25 ns simulation, the unbound had the most notable changes with one of the NAC domains (cyan), which kinked in two places, and with the C-termini, where one (green) became detached from the others and linear (Figure 12B–D). The other C-termini stayed somewhat globular, although still disordered. After 25 ns in the presence of PBA molecules, one or two of the five proteins disassociated from the protein (Figure 12F–H). The other 3–4 were bound to the helical bundle. As with the unbound simulation, one of the chains (cyan) and the C-termini stood out; however, in the presence of PBA, it was more pronounced. The cyan chain’s NAC region completely disassociated from the rest of the bundle, while the C-termini all became detached from each other, with three adopting a linear conformation.

The unfolding of the C-terminus in the presence of PBA could explain its effect on the aggregation inhibition of toxic oligomers. The C-terminal tail has been shown to be both critical for oligomerization and inhibitory to fibrillation, and its truncation leads to a decreased lag time in fibrillation.^44^ The interactions between the anionic residues of the C-terminus and residues in the NAC and N-terminus have also been shown and are believed to protect against fibrillation.^45^ However, the C-termini in the oligomeric states form disordered globular structures that contain a hydrophobic interior and polar exterior. Thus, the C-termini may also interact with themselves to promote/enhance oligomerization and then maintain the oligomerization state by stabilizing it.^46,47^

**Figure 13:**
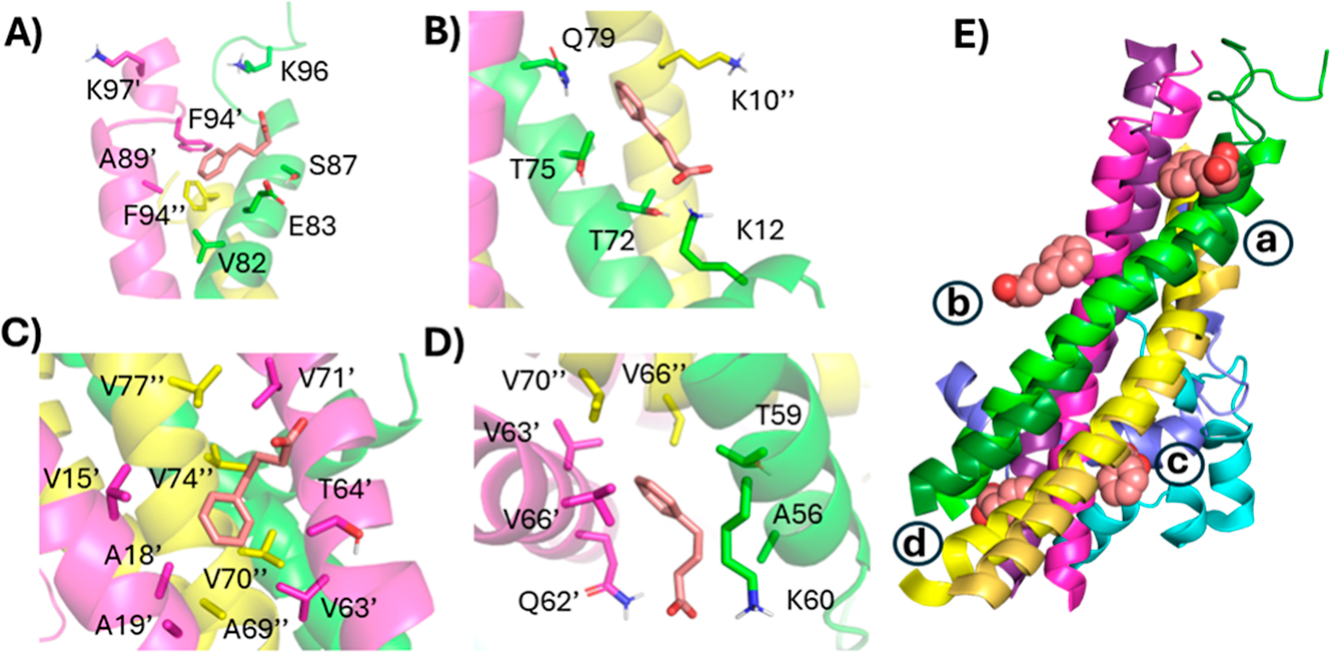
Typical binding model between PBA and tetrameric α-Syn. The binding of four PBA molecules (A–D) to tetrameric α-Syn following 25 ns MD simulation of the first replicant. Each α-Syn chain is represented as a differently colored cartoon, while PBA molecules are shown as salmon spheres. (E) The orientation between the tetra-helical bundles following 25 ns for unbound (darker shades) and PBA-bound (lighter shades) systems. Each PBA molecule is marked a–d to indicate which panel it is associated with.

Since it is immediately unclear how PBA could induce such an unfolding of the C-termini through its interaction with the NAC helical bundle, we investigated the interactions between PBA and the tetramer following a 25 ns simulation of the replicant one. The C-terminal-most PBA interacts with three chains, with the aromatic ring making potential pi–pi interactions with F94 from two different chains, and though not present at the 25 ns time point, the carboxylate spent time interacting with two lysine residues from two different chains (Figure 13A). A second molecule sits at the interface between one chain’s NAC helix and the same and another chain’s N-terminus. A shallow pocket accommodates the aromatic ring, while the carboxylate may interact with two different lysine residues (Figure 13B). Further toward the N-terminus, a third PBA molecule interacts with two chains, NAC helix and a N-terminal helix, driven by hydrophobic interactions with the aromatic ring (Figure 13C). The N-terminal-most PBA molecule docked into the intersection of three chains’ NAC helices and interacted with a hydrophobic pocket and K60 (Figure 13D). The net effect is that the individual helices are in different regions of PBA binding.

The binding of PBA is nonspecific but is modeled to occur at the interface between two or more helices containing a hydrophobic cleft and one (or more) lysine residues. Certain residues may be important for binding PBA. For example, in both the trimer and tetramer systems, PBA was modeled to interact with residues T59, K60, and Q62, sometimes with the same residue on multiple chains. In the tetrameric system where the NAC domains form a helical bundle, the C-terminal-most binding site seems worthy of further investigation. Specifically, F94 of two different chains is modeled to interact with the aromatic ring of PBA, while K96 and K97 are both modeled to interact with PBA. The binding at this site may destabilize the tetramers, which could be responsible for the observations we are seeing with PBA. Furthermore, both simulations with and without PBA in the trimer and tetrameric models show a decrease in intramolecular α-Syn hydrogen bonds, highlighting the dynamic nature of α-Syn oligomers (Figure S4). The effect of PBA on the oligomers appears to be context-dependent. The number of hydrogen bonds was generally higher in trimer systems with PBA than without, suggesting it may act to partially stabilize the trimer (Figure S4A,B). However, in the case of the tetramers with and without PBA, the effect is reversed, suggesting that it may have a destabilizing effect on the tetramer (Figure S4C,D). While assessing the GROMACS energy of the trimer, it is evident that PBA demonstrates a context-dependent α-Syn oligomer interaction with a preference to stabilize the trimer and destabilize the tetrameric oligomer, further supporting a pronounced PBA interaction and effect as α-Syn oligomerizes (Figure S5).

## Conclusions

In summary, our results provide valuable mechanistic insights into how PBA modulates α-Syn aggregation. Using a ThT assay to investigate the kinetics of this phenomenon, we observe that PBA accelerates the fibrillation of α-Syn via a reduced lag time and an early onset of the elongation phase. In addition, the ThT data indicated an increase in the kinetic rate of α-Syn fibrinogenesis, which in turn is consistent with PBA’s ability to overcome the energy barrier associated with random coil-β-rich sheet structural transition as seen in CD studies. At higher PBA concentrations, the route of this inhibition skips an intermediary transition associated with the transformation of oligomeric aggregates to high-order aggregates. As the population of α-Syn oligomers is significantly reduced, an oligomeric-mediated inhibition further suggests a decrease in amyloid-induced neurotoxicity. Our results indicate a significant reduction in cell cytotoxicity and, more importantly, an increased susceptibility of α-Syn oligomers to proteolytic cleavage under the influence of PBA. This inhibitory effect on oligomeric intermediates implies a regulatory role for PBA in preventing the early stages of α-Syn aggregation and a susceptibility for targeting α-Syn aggregation. Nile red, Congo red, and SPR binding assays indicate preferential binding interaction between PBA and hydrophobic contacts on α-Syn aggregates. Both the AFM results and the ThT microscopy reveal PBA’s ability to induce cross-linking of fibrillar aggregates of α-Syn. This phenomenon leads to the formation of longer and larger fibrils and sheds light on a potential mechanism by which PBA may modulate the structural characteristics of α-Syn aggregates. In support of the above, the DLS results suggest PBA induces the formation of α-Syn aggregates of higher hydrodynamic radii and polydispersity, indicative of the different extent of cross-linking among fibrils. Our in-silico experiments which were modeled to investigate PBA interaction with α-Syn oligomers further augment our experimental results and elucidate how PBA interactions with hydrophobic residues in the NAC region ultimately result in the destabilization of the C-terminus. We believe that such interactions mediate the increased aggregation propensity in the presence of PBA. Overall, PBA’s modulation of α-Syn fibrillation may pave the way for targeting the aberrant aggregation of other proteins that have been implicated in neurodegenerative disorders.

## Methods

### Materials and Instrumentation

Chemicals and Reagents: PBA (chemical structure: Figure S11) was obtained from Sigma-Aldrich (St. Louis, MO). Other reagent-grade chemicals including glycine, thioflavin T, Congo red, Nile red, and 2, 5-diphenyltetrazolium bromide (MTT), utilized in this investigation were procured from ThermoFisher Scientific (Waltham, MA)-verified vendors. A Milli-Q system (Millipore Corp., Bedford, MA) was used to deionize and doubly distill water. Fluorescence assays and UV–vis experiments were performed using a Spectramax M5 plate reader. Fluorescence microscopy experiments were performed using an Olympus IX73 fluorescence microscope. AFM images were obtained using Bruker Dimension Icon AFM. A Jasco J-815 CD spectrometer and Reichert’s SR7500 DC SPR system with a dual channel were used to conduct circular dichroism (CD) and surface plasmon resonance (SPR) experiments, respectively.

### Protein Expression

A DE3 expression plasmid containing human a-Syn cDNA was obtained from Addgene (pET21a-alpha-synuclein was a gift from the Michael J Fox Foundation, MJFF) [Addgene plasmid no. 51486; http://n2t.net/addgene:51486; RRID: Addgene was transformed into *E. coli* BL21 (DE3) Star (ThermoFisher Scientific)]. The cells were placed in a hot water bath at 42 °C for 45 s and placed back in the ice for 5 min intermittently. The cells were inoculated into 950 μL of LB broth with SOC media (Sigma-Aldrich) and incubated at 37 °C. The cells were spread on LB agar plates and left to grow for 48 h. Following transformation (Figure S6), on day 2, one colony was transferred into 20 mL of a Luria broth (LB) medium with ampicillin at a 100 mg/mL concentration to make the starter culture. The starter culture was kept on a shaker at 37 °C and at a low rpm of 200 and left overnight. Once the OD_600_ reached 1.8, 20 mL of the starter culture was added to 1.0 L of sterile LB medium. The concentration of the antibiotics was adjusted to 100 mg/mL. The culture was then grown at 37 °C with agitation at 200 rpm on a shaker. At an OD_600_ of ∼0.6, the cells were induced with 1000 μL of 1 M isopropyl β-thiogalactopyranoside (IPTG). The culture was grown for an additional 6 h before harvesting the cell pellets.

### Protein Purification

IPTG-induced bacterial cells were centrifuged to form a pellet, which was then resuspended in a buffer solution (comprising 50 mM Tris, pH 8.0, 10 mM EDTA, and 150 mM NaCl) containing a protease inhibitor cocktail. Subsequently, the resuspended mixture was sonicated and heated in a boiling water bath for 20 min. The resulting supernatant was isolated after centrifugation (14,000*g*, 30 min). Streptomycin sulfate (10%; 136 μL/mL supernatant) and glacial acetic acid (228 μL/mL supernatant) were added to the supernatant, and the mixture was subjected to further centrifugation (14,000*g*, 4 °C, 10 min). The obtained supernatant was precipitated by adding an equal volume of saturated ammonium sulfate maintained at 4 °C. The precipitated protein was washed using a solution of ammonium sulfate (saturated ammonium sulfate and water in a 1:1 v/v ratio at 4 °C). Precipitation was performed in three different fractions to allow for better separation of the precipitates. The washed pellet was then resuspended in 100 mM ammonium acetate and stirred for 10 min. α-Syn was precipitated again by adding an equal volume of absolute ethanol. This ethanol precipitation process was repeated four times. Finally, the protein was resuspended in 100 mM ammonium acetate, lyophilized, and stored at −20 °C until further use. The purity of α-Syn was assessed by SDS-PAGE as shown in Figure S7. The conformation of the purified protein was further investigated by using CD spectroscopy. Monomeric forms of the purified α-Syn exhibited a random coil conformation which is consistent with the literature (Figure S8).

### Preparation of Low-Molecular-Weight (LMW) α-Syn

The protein solution was subjected to dialysis overnight at 4 °C using a 10 kDa MWCO minidialysis unit (Millipore) to ensure the removal of ammonium acetate, ammonium sulfate salts, and fragmented peptides. The dialyzed protein was frozen at −80 °C, subjected to lyophilization, and stored at −20 °C for future use. To further eliminate any larger aggregates, the resulting protein solution was filtered through either a Centricon YM-100 MWCO filter (Millipore) to remove both aggregates and protein fragments to obtain monomeric α-Syn. The supernatant was collected and used for subsequent analysis.

### Amyloid Fibril Formation

The assembly reaction was initiated using α-syn at a concentration of approximately 400 μM within a 1.5 mL Eppendorf tube containing 20 mM glycine buffer. To solubilize the protein, a small volume of a 2 M NaOH solution was added incrementally until complete dissolution, and the solution became clear. Subsequently, the pH was adjusted to 6.0 by adding a 2 M HCl solution. The 1.5 mL Eppendorf tubes, each filled with protein solutions, were placed inside a thermomixer (Thermo Scientific) set at a speed of 250 rpm and at a temperature of 37 °C. The progression of fibril formation was monitored through circular dichroism (CD) and thioflavin T (ThT) binding assays, with confirmation provided by atomic force microscopy (AFM) studies.

### Circular Dichroism Spectroscopy (CD)

Fifteen microliters of 5.6 mg/mL of α-Syn were diluted to a volume of 350 μL of the glycine buffer. The resulting solution was placed in a quartz cell with a 0.1 cm path length. Spectra were acquired using a JASCO-810 instrument, with all measurements conducted at 25 °C. Spectra were recorded within the wavelength range of 198–260 nm. Five data accumulations were collected for each sample. The raw data were processed by smoothing using the Savitsky-Golay filter after subtracting buffer spectra from the sample data in accordance with the manufacturer’s instructions.

### SDS-PAGE Assay

Samples collected at important steps during the purification experiment were analyzed by using SDS-PAGE. A Laemmli protein loading buffer containing β-mercaptanol and bromophenol blue dye was added to the samples and boiled for 10 min at 100 °C. The samples were loaded onto a 15% SDS-PAGE gel after adding 2X SDS sample buffer. The experiment was run at a constant voltage of 150 V.

### Proteinase K (PK) Assay

Purified α-Syn monomers subjected to fibrillation conditions with and without PBA (1 μM, 100 μM, and 1000 μM) were collected and analyzed using the PK assay. Subsequently, the samples were subjected to proteinase K digestion (2.5 μg/mL) for 30 min at 37 °C. The reaction was stopped, and the samples were subjected to heating at 100 °C for 10 min. The samples were assessed on 15% SDS-PAGE gels after adding 2× SDS sample buffer.

### ThT Assay

To track the fibril formation of the α-Syn protein, we employed the thioflavin T (ThT) assay. ThT is a fluorescent dye known for its interaction with fibrils possessing a β-sheet structure. It exhibits enhanced fluorescence upon binding to amyloid fibrils, commonly serving as a tool to monitor amyloid fibril formation. In this study, the ThT assay was utilized to observe the impact of PBA on α-Syn aggregation. The kinetics of fibril formation in the presence of PBA was calculated by measuring the ThT fluorescent emission maximum shift to 486 nm. For each sample, 10 μL of daily fresh samples was added to 40 μL of ThT (final concentration: 5 μM α-Syn and 20 μM of ThT). Fluorescence (excitation at 450 nm and emission at 486 nm) was measured using a microplate reader with a 384-well black microwell plate. The kinetic profiles for the samples were subjected to curve fitting using a sigmoidal equation and the “least-squares” fitting method in Excel. The rate constant was determined from the generic sigmoidal equation below:

Fluorescence values were subjected to curve fitting using a sigmoidal equation through the “least-squares” fitting method in Excel. Plots were then generated to illustrate the distinct phases of the fibril growth for each sample. The employed generic sigmoidal equation for deriving kinetic parameters is as follows
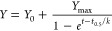
Here, *Y* represents the fluorescence intensity at various time points, while *Y*_0_ signifies the intensity of α-Syn monomers at the time point 0 min. *Y*_max_ is the maximum fluorescence emission intensity of α-Syn fibrils, *k* represents the rate constant of α-Syn aggregation, while *t*_0.5_ denotes the time required to achieve half of the maximum fluorescence intensity. This mathematical model was instrumental in characterizing the kinetics of the α-Syn fibril growth. To evaluate the seeding activity, purified monomeric α-Syn at a concentration of 370 μM was subjected to incubation at 37 °C for 120 h and an rpm of 50 using an Eppendorf thermomixer F1.5. The confirmation of amyloid formation was based on the intensity of ThT fluorescence and the presence of β-sheet secondary structure observed using CD spectra. We also confirmed the formation of fibrillar morphology of the sample using AFM. After aggregation, we subjected the sample to centrifugation at 15,000*g* and 25 °C for 30 min. To determine the concentration of the supernatant, we used a molar absorption coefficient value of α-Syn of 5960 M^–1^ cm^–1^ and performed UV absorbance of 280 nm. To generate seeds, the centrifuged fibrils were suspended in a 20 mM Gly-NaOH buffer at pH 7.6 and sonicated at 40% amplitude for 2 min intermittently. The aggregation kinetics of α-Syn were observed for 5% α-Syn seeds under the conditions of (1) the absence of PBA and (2) the presence of three different PBA concentrations, 1 μM, 100 μM, and 1000 μM. Changes in ThT fluorescence were monitored in 96-well plates (Corning, nontreated, black, clear bottom) specifically at a wavelength of 482 nm. We plotted the increase in ThT fluorescence intensity against time for all of the samples.

### Atomic Force Microscopy

Atomic force microscopy (AFM) was used to analyze morphological alterations during α-syn fibrillation with and without PBA. A 25 μM α-Syn sample was diluted to 2.5 μM, and imaging samples were prepared on mica substrates. Scans were conducted with a Bruker Dimension Icon AFM in tapping mode using an AFM silicon cantilever with HiRes150 Si tips. Intermittent washing and drying of the substrate were performed.

### Congo Red Assay

Congo red (CR) binding assay involved adding 20 μM α-Syn samples (including samples incubated with PBA) to 10 μM CR in a pH 3.0 glycine-HCl buffer, followed by a 30 min incubation. Subsequently, UV–vis absorption spectra were recorded within the 400–650 nm range. Control experiments were conducted by using α-Syn in the absence of PBA.

### Nile Red Assay

For the Nile red (NR) fluorescence assay, α-Syn samples were mixed with NR at a final concentration of 10 μM, both with and without PBA. After the addition of NR, samples were briefly incubated for 30 min. NR fluorescence was measured at an excitation wavelength of 530 nm. Emission spectra were then recorded between the wavelengths of 550–800 nm.

### Fluorescence Microscopy

Fluorescence microscopy was employed to analyze fibril morphology and distribution. α-Syn samples were incubated at pH 7 in the presence and absence of PBA. Next, 40 μM ThT solution was added to each of the samples. The fluorescence images were captured using an Olympus IX73 fluorescence microscope.

### Surface Plasmon Resonance (SPR)

Surface plasmon resonance (SPR) experiments measured the interaction between PBA and α-Syn at 25 °C. The CM5 sensor chip was activated, and the α-Syn monomer and fibrils were immobilized on the chip. The different PBA concentrations with varied surface charges were injected at a concentration of 0.2 nM. The sensor surface was regenerated after each analyte run.

### MTT Assay

The MTT assay was employed to assess the cytotoxicity of α-Syn aggregates and fibrils in the presence and absence of PBA using the SH-SY5Y cell line. Cells were plated in 96-well plates at a density of 2500 cells per well and allowed to adhere for 24 h. After 24 h, the cells were treated with α-Syn fibrils generated in the presence and absence of PBA. The cells were kept overnight at 37 °C in a humidified incubator with 5% CO_2_ to allow adherence and growth. Following treatment, the culture medium was removed. The MTT solution (0.5 mg/mL in PBS) was added to each well followed by incubation for 4 to 6 h at 37 °C. Subsequently, the MTT solution was aspirated, and formazan crystals formed by metabolically active cells were solubilized with acidic isopropyl alcohol (containing 0.04 N HCl). The plates were gently mixed to ensure thorough solubilization. The absorbance of the solubilized formazan solution in each well was measured at 560 nm by using a microplate reader. Background absorbance was subtracted from the absorbance at 560 nm to correct for interference. The assay was conducted in triplicate to ensure consistency and reliability of the results.

### Dynamic Light Scattering

Dynamic light scattering (DLS) experiments were conducted using a Zetasizer Nano ZS (Malvern Nano ZS) instrument equipped with a 4 mW He–Ne laser beam and a Peltier temperature controller. Using disposable microcuvettes, DLS assessments of various α-Syn fibrillar samples (5 μM) in both the presence and absence of PBA were performed at a wavelength of λ = 632.5 nm and a scattering angle of 173°. Measurements for each sample were performed three times, and the experiment was done in a thermostatic sample chamber set at 37 °C. The averaged intensity-size distribution for the different samples was reported.

### Statistical Data Analysis

All the experimental data reported in this work were obtained from three replicates. For ThT experiments, values are expressed as mean ± SD. For DLS measurements, values are given as mean ± SD (*n* = 3 from independent experiments). AFM, fluorescence, SDS-PAGE, and proteinase K were repeated thrice. MTT assay data is represented as means ± SD from the average of 3 wells. Statistical data analysis for cell viability assay was determined using GraphPad Prism software. One-way ANOVA and Dunnett’s post-hoc test were used for multiple comparisons with 95% confidence intervals, where *P < 0.05 and ***P < 0.001 indicate statistically significant differences.

### Molecular Dynamics Simulations

A representative helical-rich trimer (as multi-300) and tetramer structure (as multi-238) was selected from a set of trimeric and tetrameric α-Syn oligomers developed by Gurry et al.^43^ PBA was globally docked to each rigid oligomer 1000 times using Autodock Vina. The grid sizes chosen were 84 × 100 × 92 Å for the trimer and 74 × 118 × 92 Å for the tetramer. To further enhance sampling, we set the exhaustiveness to 16. Of the 10,000 poses outputted, the top thousand docked poses were mapped to the oligomer, where they clustered to 14 individual, unique sites for the trimer and four sites for the tetramer. At each site, the top-scoring PBA molecule pose was selected for use in molecular dynamics experiments. CHARMM-GUI was used to build systems of α-Syn oligomers with and without PBA.^48,49^ PBA ligands were parameterized using the CHARMM General Force Field (CGenFF) (PMID 19575467). The solvated system was neutralized with potassium atoms (27 for the unbound trimer, 41 for the trimer with 14 PBA molecules, 36 for the unbound tetramer, and 41 for the tetramer with 5 PBA molecules) into a cube (102 Å side length for the trimer and 142 Å side length for the tetramer). GROMACS was used for the molecular dynamics simulations, using the CHARMM36m force field.^50^ Each system was subjected to the steepest decent minimization and then equilibrated at 303.15 K with a 120 ps *NVT* simulation. Equilibrated systems were then subjected to a 25 ns *NPT* simulation.

## Supporting Information Available

Kinetics of α-Syn fibrillation in the absence and presence of PBA, size distributions, analysis of the secondary structure, and additional results (PDF)
