# Supplementary material for: Modulation of α-Synuclein Fibrillation and Toxicity by 4-Phenylbutyric Acid
Source: ACS Chem Neurosci. 2025 Feb 28;16(6):1066–78. doi: 10.1021/acschemneuro.4c00709 (PMC11926867; doi:10.1021/acschemneuro.4c00709)
Supplement: Supplementary file 1 — cn4c00709_si_001.pdf [file cn4c00709_si_001.pdf]

"Supporting Information"

Modulation of α-Synuclein Fibrillation and Toxicity by 4-Phenylbutyric Acid

Kristos Baffour,[1] Neelima Koti,[1] Tony Nyabayo,[1] Sathvika Balerao,[1] Carissa Sutton,[1] David Johnson,[2] Rishi Patel,[3] Santimukul Santra,[1] and Tuhina Banerjee[1,]*

[1]Department of Chemistry and Biochemistry, Missouri State University, 901 S. National Avenue, Springfield, MO 65897, United States of America

[2]Molecular Graphics and Modeling Laboratory, University of Kansas, 2034 Becker Drive, Lawrence, KS 66018, United States of America

[3]Jordan Valley Innovation Center, Missouri State University, 542 N. Boonville Avenue, Springfield, MO 65806, United States of America

*Corresponding author: Tuhina Banerjee, Email: tbanerjee@missouristate.edu

**Parameter (h)**	**α-synuclein**	**α-synuclein + 1 μM PBA**	**α-synuclein + 100 μM PBA**	**α-synuclein + 1000 μM PBA**
$t_{1/2}$	41.4 ± 6.5	28.2 ± 3.0	8.3 ± 3.0	5.8 ± 0.8
Lag time	52.1 ± 5.0	41.5 ± 3.1	26.7 ± 3.5	21.5 ± 1.0

Table S1: Kinetics of α-Syn fibrillation in the absence and presence of different concentrations of PBA. Results indicate PBA accelerates fibril formation by reducing the lag phase.

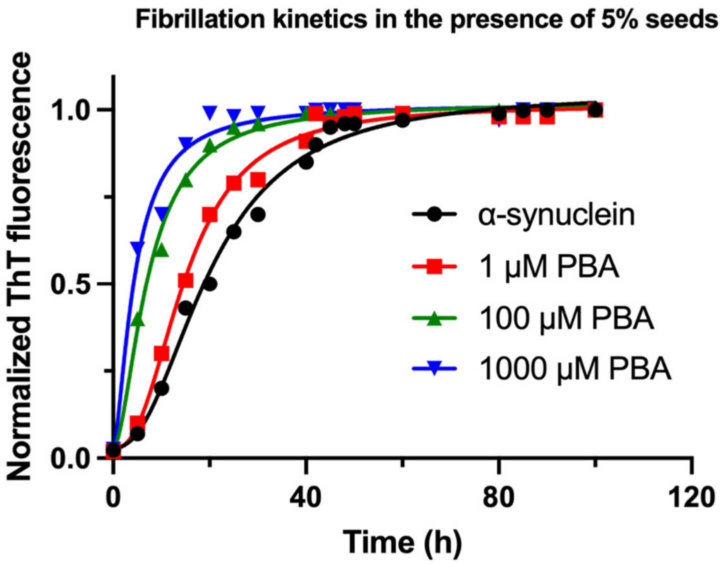

Figure S1: Aggregation kinetics profile of α-Syn in the presence of 5% seeds generated with increasing concentrations of PBA.

Experimental condition	Size
**α-Syn only**	55 nm,1060 nm
**α-Syn + 1 μM PBA**	90 nm,1280 nm
**α-Syn + 100 μM PBA**	1290 nm
**α-Syn + 1000 μM PBA**	1320 nm

Table S2: Representative data for the size of the α-Syn during fibrillation in the presence of different concentrations of PBA using Dynamic Light Scattering Studies.

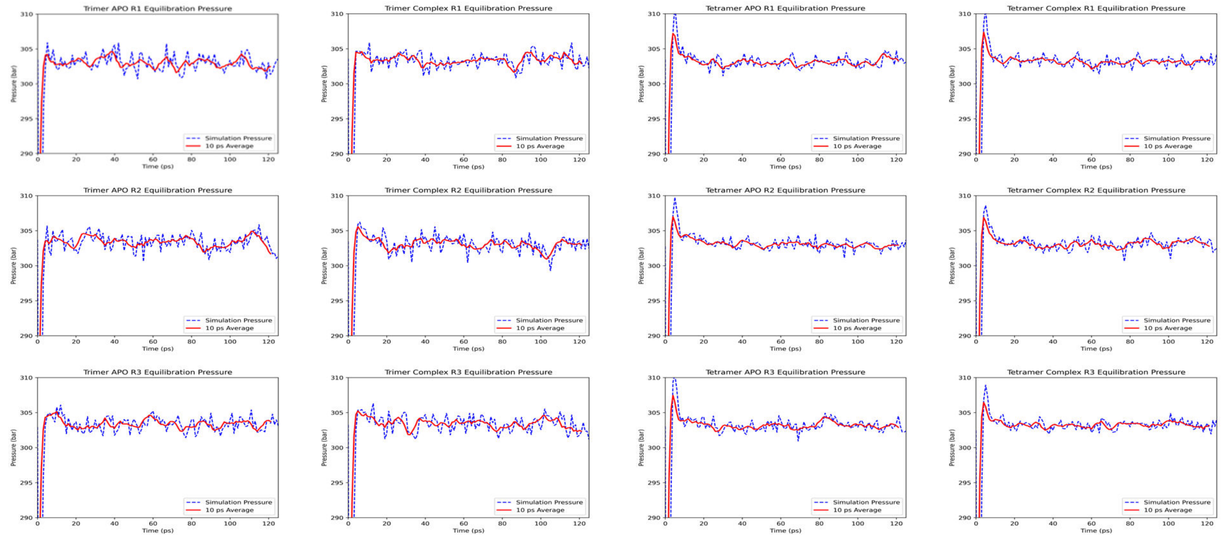

Figure S2: Pressure is stable during the NVT equilibration. The pressure of the system is shown in blue for the equilibration run for each replicant of each system. The 10 ps running average is shown in red.

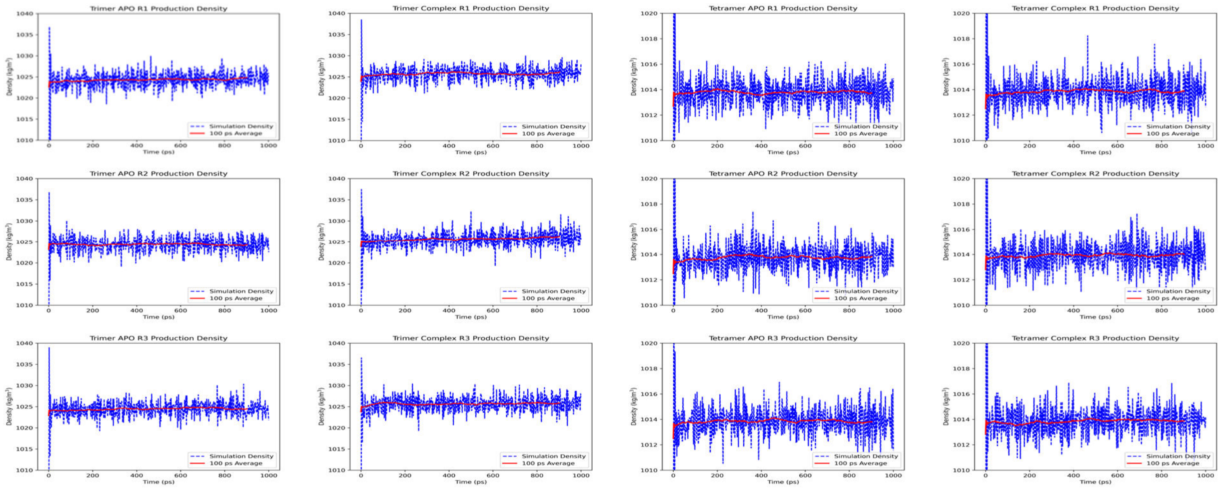

Figure S3: Density rapidly stabilizes during production simulation. The density of the system is shown in blue for the first nanosecond of the production run for each replicant of each system. The 100 ps running average is shown in red.

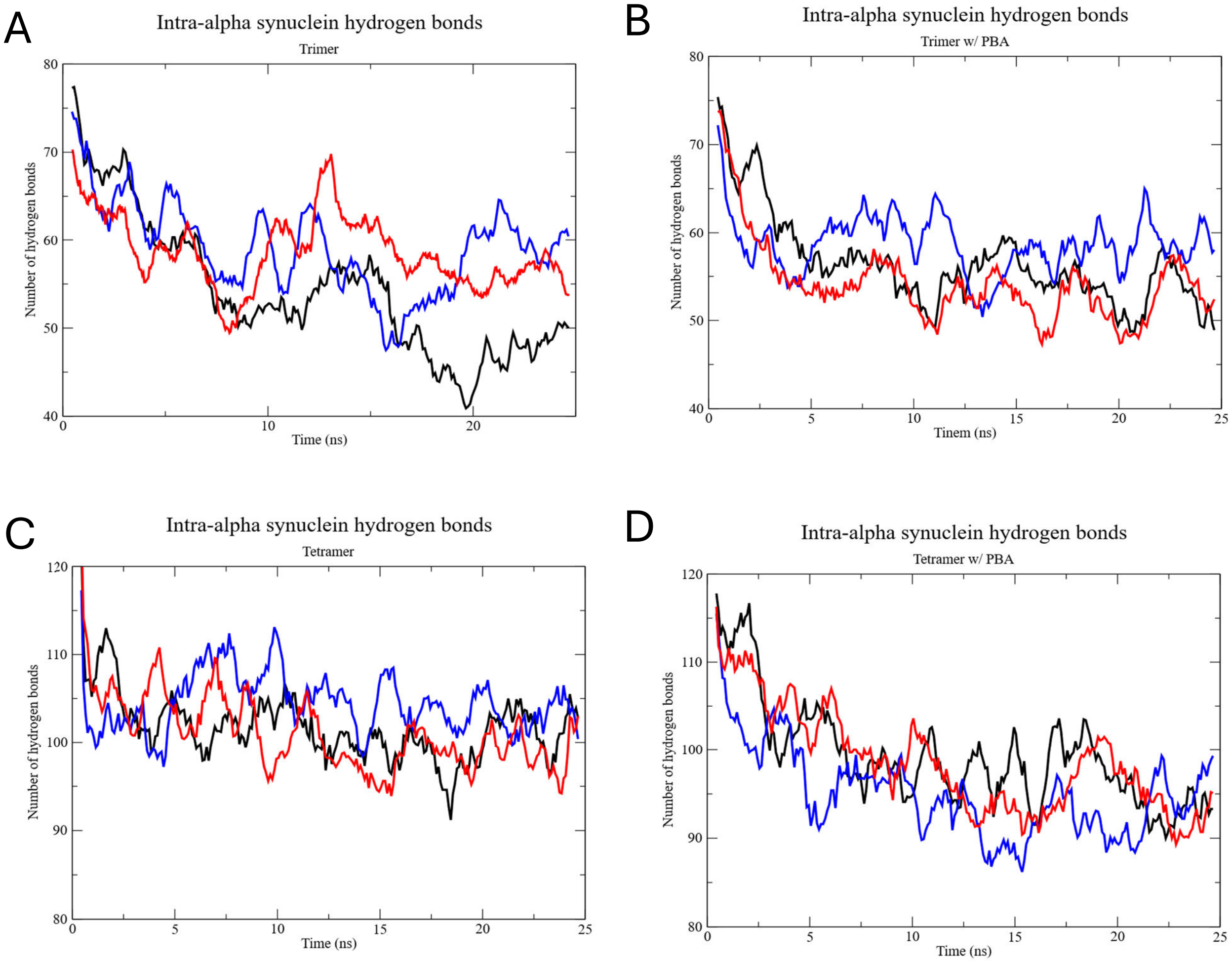

Figure S4: Number of α-Syn hydrogen bonds throughout the course of the (MD) simulation. The 1 ns running average of hydrogen bonds within α-Syn for the (**A and B**) trimer without and with PBA (**C and D**) tetramer without and with PBA are plotted against time. Replicants one, two, and three are colored black, blue, and red, respectively.

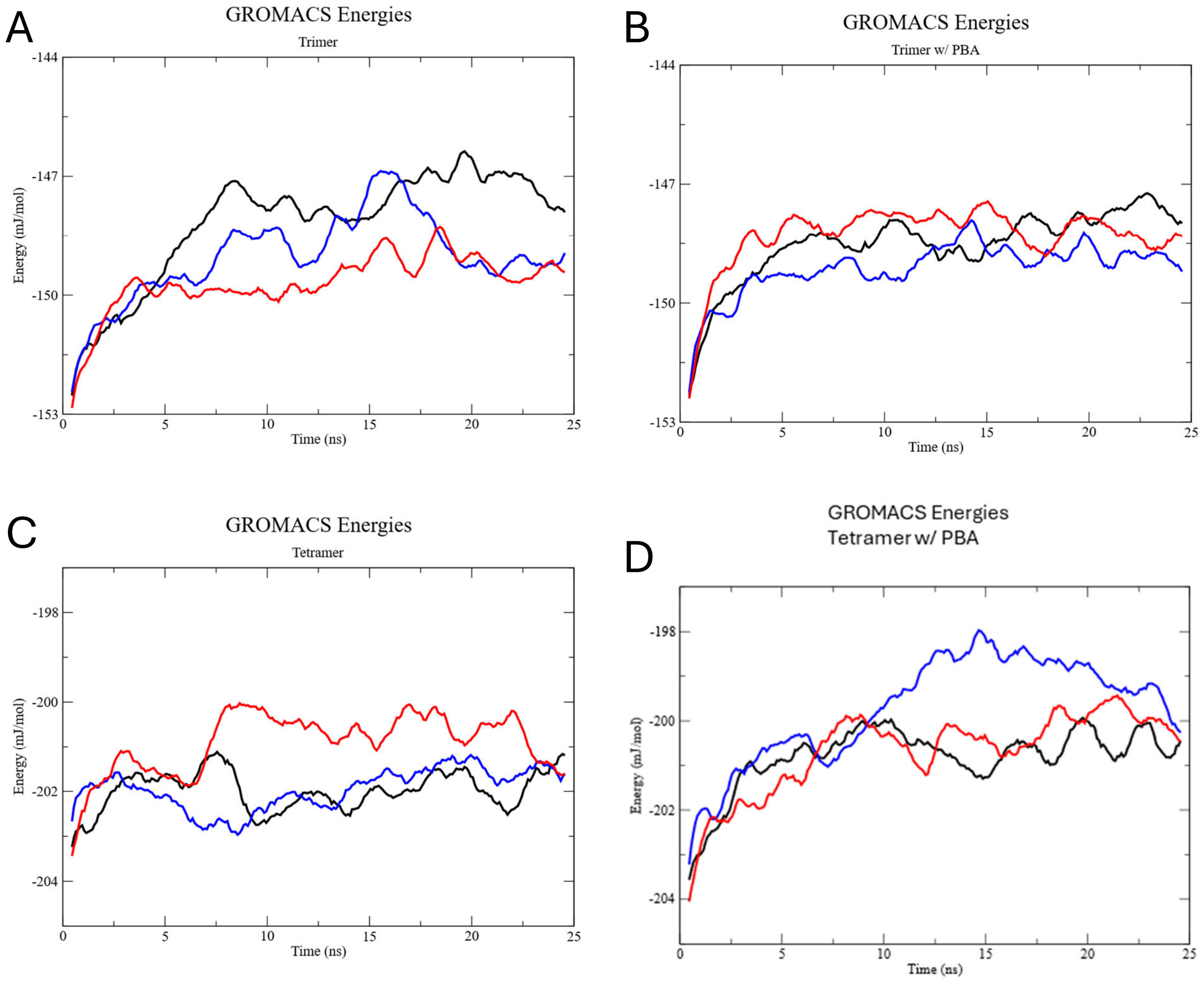

Figure S5: Rough approximations of the energies of α-Syn, over time. The sum of the short-range Lennard-Jones and coulombic potentials for the α-Syn (**A and B**) trimer without and with PBA and (**C and D**) tetramer without and with PBA are plotted against time. Replicants one, two, and three are colored black, blue, and red, respectively.

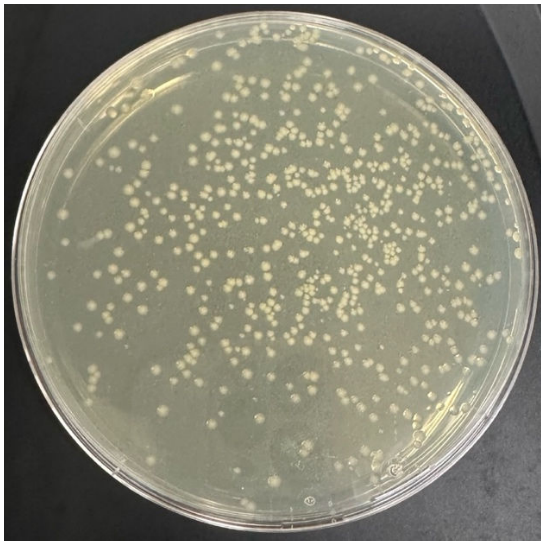

Figure S6: Isolated colonies formed after transformation of α-Syn plasmid into *E.coli* BL21 competent cells.

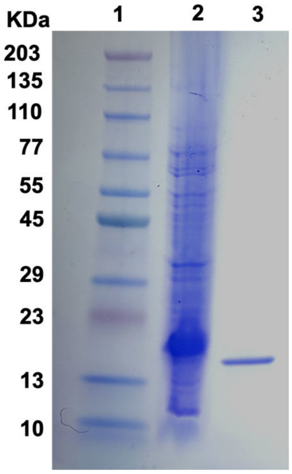

Figure S7: SDS PAGE analysis of α-Syn purification. **1**) Ladder, **2**) cell lysate of α-Syn monomer and **3**) purified monomer.

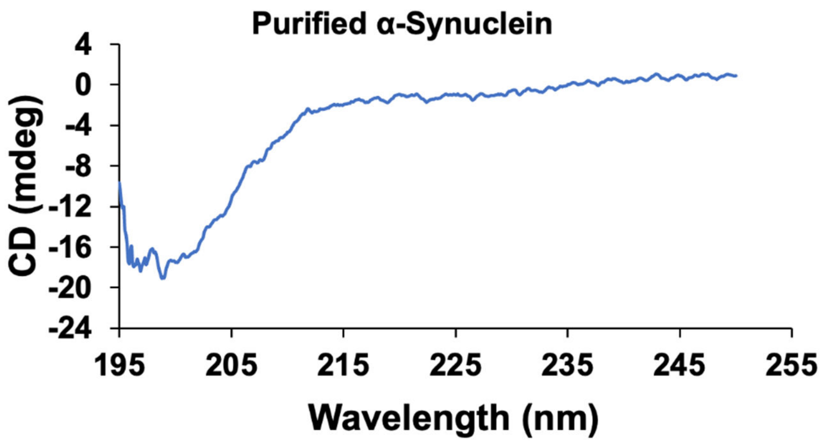

Figure S8: Far-UV CD spectra of purified α-synuclein.

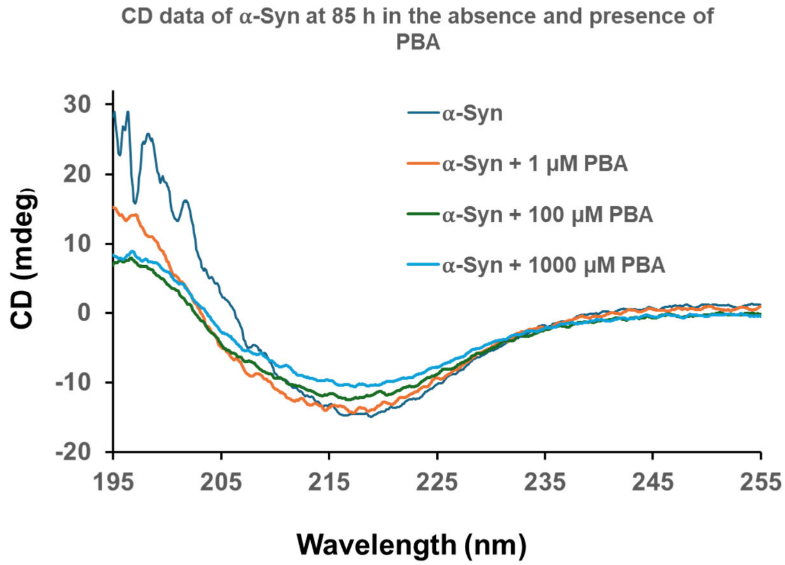

Figure S9: Overlay of far UV-CD spectra of α-Syn at 85 h in the absence and presence of PBA.

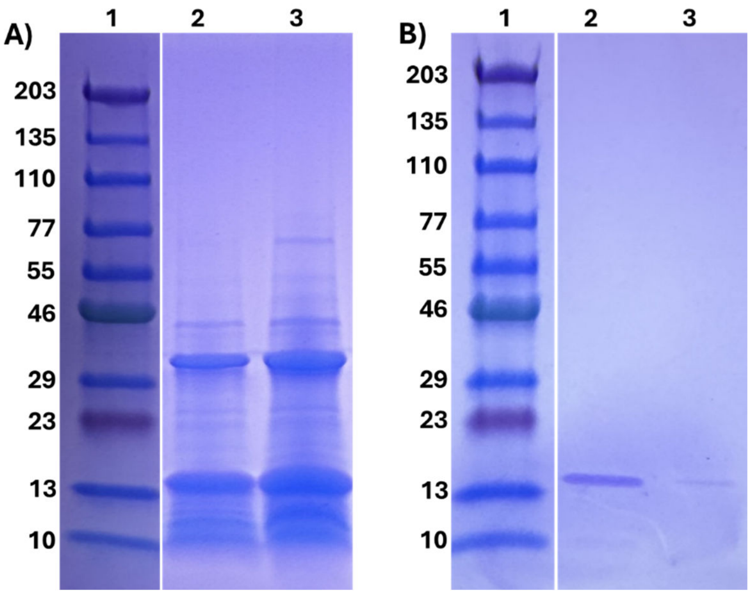

Figure S10: SDS-PAGE analysis of the total α-Syn fibrils in the absence and presence of 1000 μM PBA at time point 85 h following ultracentrifugation at 100,000 g for 30 min at 4°C. A) pellet fractions: obtained with α-Syn only (lane 2) and α-Syn + 1000 1000 μM PBA (lane 3). B) soluble supernatant fraction obtained with α-Syn only (lane 2) and α-Syn + 1000 μM PBA (lane 3). Marker has been shown for comparison.

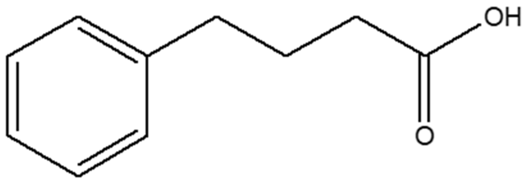

4-Phenylbutyric acid (PBA)

Figure S11: Chemical structure of PBA.

A)

**Secondary Structure**	**α-Syn (0 h), %**	**α-Syn + I μM PBA (0 h), %**	**α-Syn + 100 μM PBA (0 h), %**	**α-Syn + 1000μM PBA (0 h), %**
**Helix**	0	0	0	0
**Antiparallel**	31.4	28.8	27.2	25.5
**Parallel**	0	0	0	0
**Turns**	19.7	21.6	23.4	26.6
**Others**	48.9	49.6	49.4	47.9

B)

**Secondary Structure**	**α-Syn (85 h), %**	**α-Syn + I μM PBA (85 h), %**	**α-Syn + 100 μM PBA (85 h), %**	**α-Syn + 1000μM PBA (85 h), %**
**Helix**	11.0	10.2	3.2	2.7
**Antiparallel**	41.4	37.8	11.8	11.5
**Parallel**	18.0	18.6	49.9	52.0
**Turns**	3.5	7.8	11.2	8.8
**Others**	26.1	25.6	23.9	25.0

Table S3: Secondary structure estimation from Far-UV CD spectra of α-synuclein in the absence and presence of different concentrations of PBA using BeStSel at **A)** 0 h and **B)** 85 h.
